# Repeated stress to the skin amplifies neutrophil infiltration in a keratin 17- and PKCα-dependent manner

**DOI:** 10.1371/journal.pbio.3002779

**Published:** 2024-08-19

**Authors:** Yang Xu, Erez Cohen, Craig N. Johnson, Carole A. Parent, Pierre A. Coulombe

**Affiliations:** 1 Graduate Program in Pharmacology and Department of Pharmacology, University of Michigan Medical School, Ann Arbor, Michigan, United States of America; 2 Department of Cell & Developmental Biology, University of Michigan Medical School, Ann Arbor, Michigan, United States of America; 3 Life Science Institute, University of Michigan, Ann Arbor, Michigan, United States of America; 4 Rogel Cancer Center, University of Michigan Medical School, Ann Arbor, Michigan, United States of America; 5 Department of Dermatology, University of Michigan Medical School, Ann Arbor, Michigan, United States of America; Institute for Stem Cell Science and Regenerative Medicine, INDIA

## Abstract

Neutrophils are the first immune cells to reach inflamed sites and contribute to the pathogenesis of chronic inflammatory skin diseases. Yet, little is known about the pattern of neutrophil infiltration in inflamed skin in vivo and the mechanisms mediating their recruitment. Here, we provide insight into the dynamics of neutrophil infiltration in skin in response to acute or repeated inflammatory stress, highlighting a novel keratinocyte- and keratin 17 (K17)-dependent mechanism that regulates neutrophil recruitment to inflamed skin. We used the phorbol ester TPA and UVB, alone or in combination, to induce sterile inflammation in mouse skin. A single TPA treatment results in a neutrophil influx in the dermis that peaks at 12 h and resolves within 24 h. A subsequent TPA treatment or a UVB challenge, when applied 24 h but not 48 h later, accelerates, amplifies, and prolongs neutrophil infiltration. This transient amplification response (TAR) is mediated by local signals in inflamed skin, can be recapitulated in *ex vivo* culture, and involves the K17-dependent sustainment of protein kinase Cα (PKCα) activity and release of chemoattractants by stressed keratinocytes. K17 binds RACK1, a scaffold protein essential for PKCα activity. The N-terminal head domain of K17 is crucial for its association with RACK1 and regulation of PKCα activity. Analysis of RNAseq data reveals a signature consistent with TAR and PKCα activation in inflammatory skin diseases. These findings uncover a novel, keratin-dependent mechanism that amplifies neutrophil recruitment in skin under stress, with direct implications for inflammatory skin disorders.

## Abstract 2

Neutrophils contribute to the pathogenesis of chronic inflammatory diseases, but the mechanisms underlying their recruitment to inflamed skin are unclear. This study shows that repeated acute stress to the skin amplifies a neutrophil infiltration response involving keratin 17 and PKCα.

## Introduction

Inflammation and dysregulated immune responses are significant determinants of several skin-associated diseases, e.g., psoriasis (PSOR) [1,2], atopic dermatitis (AD) [1], acne [3], and hidradenitis suppurativa (HS) [4,5]. Neutrophils normally act as essential effectors of innate immunity at sites of tissue injury, infection, and inflammation [6]—however, excessive neutrophil infiltration drives the progression and severity of skin diseases through various mechanisms [2,7]. Circulating neutrophils are recruited to sites of inflammation after sensing chemical cues in a process called chemotaxis [6]. They are the first leukocytes to reach sites of tissue damage, infection, or stress, and are tasked with defending the host by releasing proteases and reactive oxygen species, phagocytosing pathogens and cell debris, and releasing genomic DNA-histone complexes known as neutrophil extracellular traps [6]. Neutrophils rarely occur in healthy skin, but readily infiltrate when skin and other tissues are subjected to environmental stresses, and in various inflammatory skin disorders including PSOR [1,2], HS [4,5], and cancer [7]. Yet, the kinetics of neutrophil infiltration and resolution and the mechanisms by which stressed skin tissue generates neutrophil-recruiting signals remain poorly understood.

Keratinocytes, the predominant cell type in the epidermis, are an important source of signals that regulate inflammation and immunity in skin [1,8]. These cells express abundant amounts of keratin proteins in a tightly regulated fashion [9]. A distinct subset of keratins, including the type II keratins K6A-C paralogs and type I K16 and K17, are prominently involved in the response of epidermis to various stresses [10,11]. Expression of K6A-C, K16, and K17 occurs in ectoderm-derived epithelial appendages and in the thick epidermis of palms and soles in healthy human and mouse skin [12]. Though absent from healthy interfollicular epidermis, K6A-C, K16, and K17 are rapidly induced after injury and many other stresses to the skin [11,13], in chronic diseases such as PSOR [10], AD [14], acne [15], and in multiple types of carcinomas [16,17]. Besides, missense mutations in any one of the genes coding for K6A-C, K16, and K17 can cause pachyonychia congenita (PC) [18,19], a rare disorder characterized by dystrophic nails, oral lesions, cutaneous cysts as well as painful and debilitating palmoplantar keratoderma. The latter are lesions driven by dysregulated epithelial differentiation, redox balance, innate immunity, and inflammatory pathways [20]. As is the case for most intermediate filament (IF) proteins, the stress-responsive keratins fulfill classic roles for cytoskeletal proteins along with noncanonical roles in the cell. In a context-dependent fashion, for example, the cytoplasmic pool of K17 regulates protein synthesis and keratinocyte growth [21,22], TNFα-induced apoptosis [22], and inflammatory/immune gene expression [23], while a small nuclear pool of K17 regulates nuclear architecture [24], gene expression [25,26], the cell cycle [24,27], and the DNA damage and repair response [28]. Though available evidence supports a role for K17 in altering inflammation and immune responses in skin experiencing stress [25,29], whether it regulates neutrophil infiltration, specifically, and the potential relevance for inflammatory skin diseases, are unknown.

Here, we show that the skin transiently adapts to a recent stress exposure and exhibits a faster, stronger, and prolonged neutrophil infiltration in response to additional stress exposures. This phenomenon, which we designate as TAR for transient amplification response, is short-lived and mediated by local, keratinocyte-derived signals. TAR entails a K17-dependent sustainment of the activity of protein kinase C alpha (PKCα), resulting in neutrophil chemokine release by stressed keratinocytes. Comparative analysis of transcriptomic data sets suggests that TAR, as observed in mouse skin under acute stress, is relevant to human inflammatory skin diseases.

## Results

### Transient amplification of neutrophil infiltration upon repeated stress to skin

**Fig 1:**
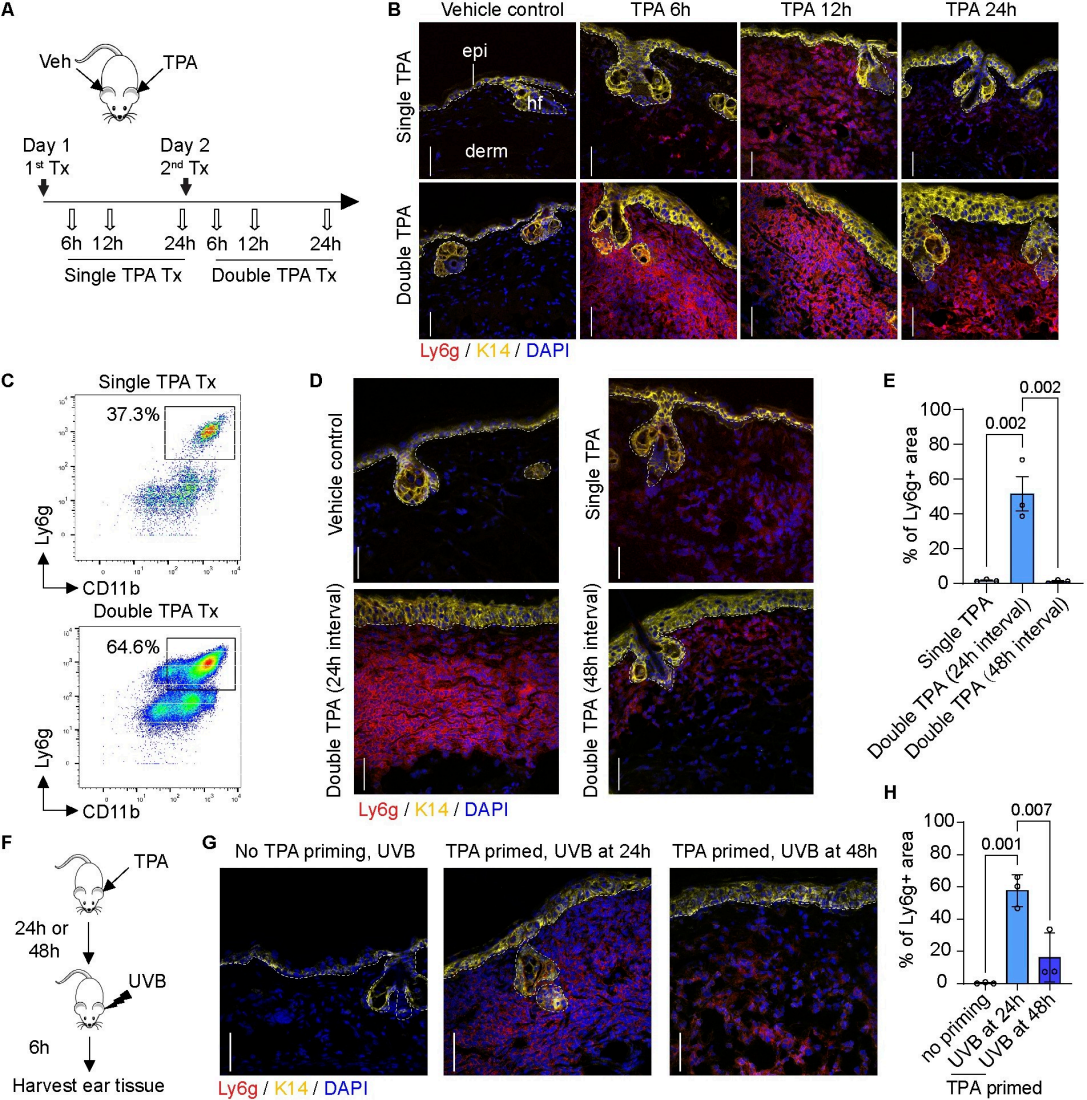
TAR in skin subjected to repeated irritation. **(A)** Strategy for single and double topical TPA Tx, 24 h apart, and skin tissue sampling. Clipart is open source https://openclipart.org/detail/17622/simple-cartoon-mouse-1; Creative Commons CC0 1.0 Universal License (https://creativecommons.org/publicdomain/zero/1.0/). **(B)** Sections of WT mouse skin treated as shown in A were immunostained for K14, Ly6g, and nuclei (DAPI). epi, epidermis; derm, dermis; hf, hair follicle. Scale bars: 50 μm. Dashed lines depict the dermo-epidermal interface. **(C)** CyTOF analysis for CD11b (granulocytes, X-axis) and Ly6g (neutrophils; Y-axis) 12 h after a single and double TPA Tx of ear skin. Cells were pre-gated as single, live, non-beads, DNA- and CD45-positive. The Ly6g+CD11b+ neutrophil populations are boxed and their frequencies indicated. *n* = 3 mice. **(D)** WT mouse skin treated with acetone, single TPA, dual TPA (24 h apart) or double TPA (48 h apart). Skin sections were immunostained for K14, Ly6g, and nuclei (DAPI). Scale bars: 50 μm. **(E)** Quantitation of neutrophil fluorescence signal (percentage of Ly6G+ surface area in total dermis) from (D). *n* = 3 mice. Data are shown as mean ± SEM. One-way ANOVA. **(F)** Strategy for UVB and TPA combination Tx and skin tissue sampling. **(G)** Sections of WT mouse skin treated as shown in F were immunostained for K14, Ly6g, and nuclei (DAPI). Scale bars: 50 μm. **(H)** Quantitation of neutrophil fluorescence signal (surface area measurements) of Ly6g from (G). *n* = 3 mice. Data are shown as mean ± SEM. One-way ANOVA. The source data used to derive the numerical values reported here can be found in S1 Data.

Mouse ear skin was treated with 12-O-tetradecanoylphorbol-13-acetate (TPA), a phorbol ester widely used to induce sterile inflammation or as a tumor promoter [30], harvested at specific time points after single or dual topical TPA treatment (Tx) (**Fig 1A**), and analyzed using immunostaining and CyTOF. Neutrophils are not present in skin at baseline (under no Tx), and topical application of the acetone vehicle control does not induce neutrophil infiltration in ear skin (S**1A Fig**). After a single topical exposure to TPA, however, a neutrophil infiltration occurs in the dermis starting at 6 h, peaks at 12 h, and is cleared by 24 h (**Fig 1B**). When a second TPA Tx is given 24 h later, the resulting neutrophil influx is significantly accelerated, amplified, and persists longer (**Fig 1B**). CyTOF analysis of immune cells isolated from mouse ear tissue confirms the large increase in CD11b+Ly6g+ neutrophil infiltration at 12 h after a second TPA Tx, compared to 12 h after a first Tx (**Fig 1C**). We next tested whether the amplification of neutrophil infiltration represents a stably acquired property in TPA-Tx skin. When the second TPA Tx is given at 48 h instead of 24 h after the first Tx, the kinetics and amplitude of neutrophil recruitment replicate the response after a single Tx–i.e., there is no amplification (**Fig 1D and 1E**). We refer to this phenomenon as a TAR whereby an earlier onset and more robust infiltration of neutrophils occurs upon repeated topical exposure to the chemical irritant TPA in a manner that depends upon the length of interval between treatments.

To assess whether TAR can be triggered by stressors other than TPA, we next subjected the mice to UVB stimulation. We found that TAR similarly takes place when the second external challenge to TPA-primed skin consists of an exposure to 400 mJ/cm^2^ UVB (**Fig 1F**). Again, we found that the response to UVB as a second treatment entails an acceleration and increased amplitude of neutrophils in the dermis, and is manifested when UVB is administered at 24 h, but not 48 h, after the initial TPA Tx (**Fig 1G and 1H**). Single or dual exposures of naïve skin to 400 mJ/cm^2^ UVB radiation does not cause a significant infiltration of neutrophils (see **Fig 1G**). The latter is an indication that while TAR is not specific to TPA-induced irritation, it requires the presence of a component that is specifically induced by acute exposure to TPA, but not UVB, in mouse skin.

### TAR is mediated by local signals and is not related to the barrier status in stressed skin

To assess whether TAR is related to the skin barrier status, we measured trans-epidermal water loss (TEWL) in dorsal ear skin treated with TPA. Significant and comparable increases in TEWL values occur at 24 h and 48 h after a single TPA Tx (**S1B Fig**), suggesting that differences in the barrier status do not account for the difference seen in neutrophil infiltration when a repeat challenge is given at 48 h versus 24 h after the initial insult. To examine whether TAR represents a systemic response after the initial stress, we performed studies in which sequential TPA Tx, 24 h apart, are applied on the same versus contralateral ears (**S1C Fig**). We found that TAR only occurs when the same ear is exposed to dual TPA Tx (**S1D and S1E Fig**). These findings show that TAR is primarily driven by local signal(s) in treated skin and cannot be transferred by systemic circulating factors to naïve sites.

### Induced K17 in keratinocytes promotes neutrophil recruitment in stressed skin

**Fig 2:**
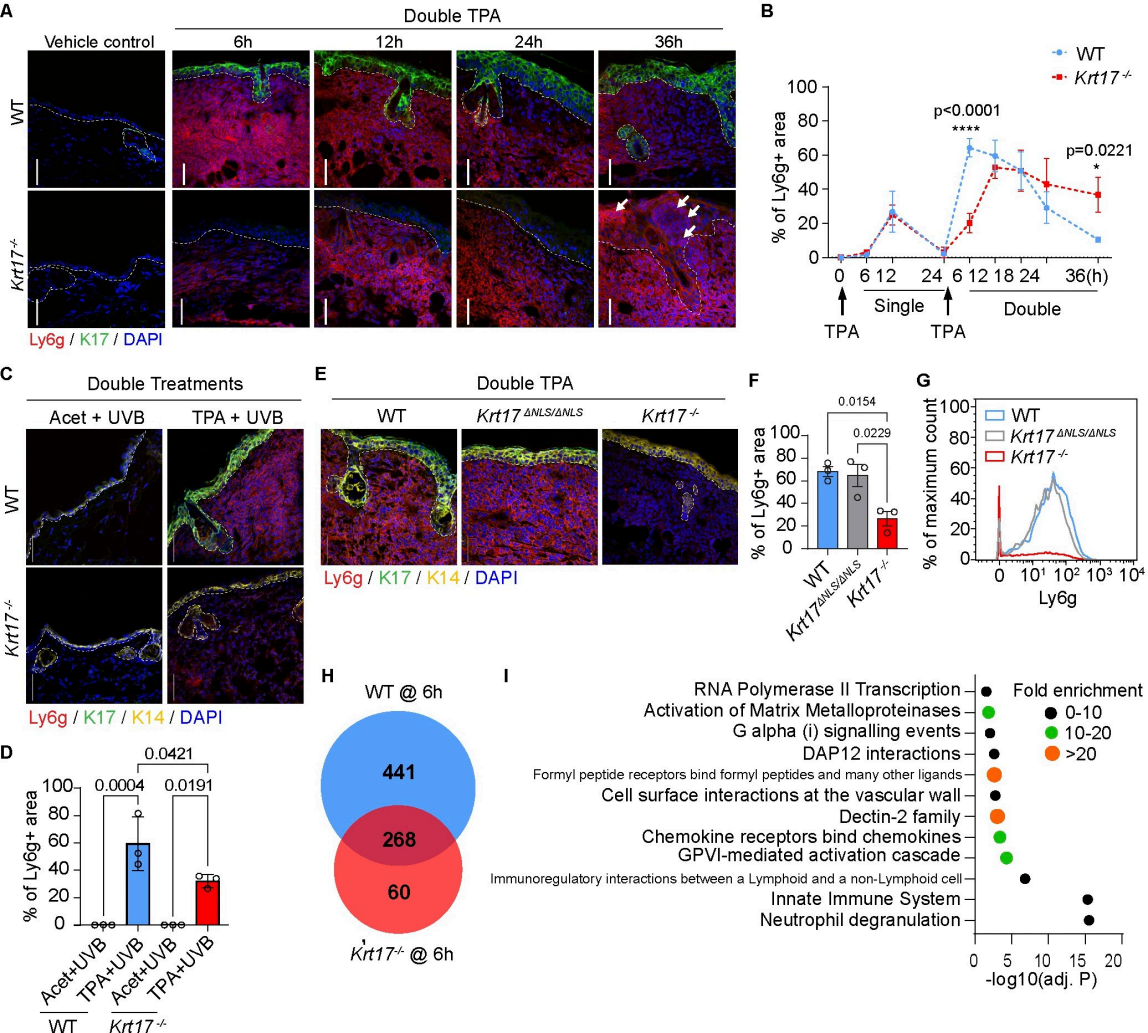
Role of induced K17 in epidermis of skin subjected to repeat topical challenges. **(A)** Sections of WT and *Krt17*^*-/-*^ mouse skin treated with double TPA, 24 h apart, immunostained stained for K14, Ly6g, and nuclei (DAPI). Time of sampling after treatment indicated above micrographs. Vehicle control images are ears treated with double acetone and harvested 6 h after the second Tx. Dashed lines depict the dermo-epidermal interface. Scale bars: 50 μm. **(B)** Quantitation of neutrophil fluorescence (surface area measurements), comparing WT (blue trace) and *Krt17*^*-/-*^ (red trace) as a function of time after a single and double TPA Tx. *n* = 3–4 mice. Data are shown as mean ± SEM. Two-way ANOVA. **(C)** Sections of WT and *Krt17*^*-/-*^ mouse skin treated with acetone or TPA, followed by UVB Tx 24 h later, were immunostained for K14, K17, Ly6g, and nuclei (DAPI). Scale bars: 50 μm. **(D)** Quantitation of neutrophil fluorescence signal (surface area measurements) from (C). *n* = 3 mice. Data are shown as mean ± SEM. One-way ANOVA. **(E)** Sections of WT, *Krt17*^*-/-*^, and *Krt17*^*ΔNLS/ΔNLS*^ mouse skin treated with double TPA, 24 h apart, were immunostained for K14, K17, Ly6g, and nuclei (DAPI). Scale bars: 50 μm. **(F)** Quantitation of neutrophil fluorescence (surface area measurements) from (E). *n* = 3 mice. Data are shown as mean ± SEM. One-way ANOVA. **(G)** CyTOF analysis for Ly6g (neutrophils) 6 h after double TPA Tx (24 h apart) of mouse ear skin. Cells were pre-gated as single, live, non-beads, DNA- and CD45-positive. The frequencies of Ly6g+ neutrophil populations are indicated. *n* = 3 mice. **(H)** Genes significantly up-regulated after dual TPA Tx compared to dual acetone-Tx, 24 h apart, in WT and *Krt17*^*-/-*^ mouse skin (Cutoffs: FDR-adjusted *P* < 0.01, fold change > 8). **(I)** Panther overrepresentation test using Reactome pathways (FDR-adjusted *P* < 0.05) for 441 genes significantly up-regulated in double TPA-treated WT skin but not in Krt17^-/-^ skin. Acet, acetone. The source data used to derive the numerical values reported here can be found in S1 Data.

Stress keratins are robustly induced in epidermal keratinocytes subjected to various challenges [11,13]. K17 is of interest regarding a potential role in TAR as it has been implicated in the immune response of TPA-Tx mouse skin and in several skin cancer models [23,25,28,29]. At baseline, K17 is expressed in skin epithelial appendages (e.g., hair follicles, sebaceous, and sweat glands) but not in the interfollicular epidermis of mouse skin (**S2A Fig**). K17 is induced within hours in the upper layers of epidermis after a single topical TPA Tx and is present in significant amounts when the skin is again exposed to TPA at later times (**Figs 2A and S2A**). We next set out to assess whether TAR occurs in ear skin of *Krt17* null mice [31]. After a single TPA Tx, neutrophil infiltration parameters are indistinguishable in *Krt17* null and WT mice, suggesting that the constitutive pool of K17 in pilosebaceous units does not impact neutrophil infiltration in the dermis (**Figs 2B and S2A**). When a second TPA Tx (or UVB exposure) is given 24 h later, however, neutrophil recruitment is significantly delayed in *Krt17* null skin (**Fig 2A–2D**). We also observed that intra-epidermal neutrophil infiltration occurs in *Krt17* null epidermis, but not in WT control, at 36 h following dual TPA Tx spaced 24 h apart (see white arrows in **Fig 2A**). Of note, neutrophil infiltration in the epidermis occurs in generalized pustular PSOR [32] and in HS [5]. Albeit very interesting, this observation was not pursued further in the current study. We found no difference in the percentage of TUNEL-positive cells 6 h after dual TPA-TPA or TPA-UVB Tx, spaced 24 h apart, in *Krt17* null versus WT mouse skin (**S2B and S2C Fig**), suggesting that the delay in TAR in *Krt17* null skin is not due to differences in apoptotic cell death. These findings show that induced K17 in TPA-primed epidermal keratinocytes plays a significant role in the early amplification of the neutrophil influx induced by repeated stresses to skin.

A small pool of K17 occurs in the nucleus of tumor keratinocytes where it impacts nuclear architecture and chromatin organization [24], pro-inflammatory gene expression [25], and DNA damage and repair [28]. To assess whether the nuclear-localized pool of K17 contributes to TAR, we subjected *Krt17*^*ΔNLS/ΔNLS*^ mice, which harbor a mutated nuclear localization signal in K17 [24], to dual TPA Tx, 24 h apart. Immunofluorescence staining of tissue sections (**Fig 2E and 2F**) and CyTOF analysis of immune cell populations (**Fig 2G**) show that ear skin of *Krt17*^*ΔNLS/ΔNLS*^ mice displays a TAR that is indistinguishable from WT. Therefore, TAR is primarily regulated by the cytoplasmic pool of K17 in stressed epidermal keratinocytes.

We next performed bulk RNAseq analysis of WT and *Krt17* null mouse ear tissue obtained at 6 h after a second TPA Tx to investigate TAR-related changes in gene expression. Volcano plots highlighting genes that are up- or down-regulated by 8-fold or more (adj. *P* < 0.01) as a function of genotype (WT versus *Krt17* null) and treatment (control versus dual TPA) are shown in **S2D and S2E Fig**. We focused subsequent analyses on up-regulated genes (**Fig 2H**; see full listing in **S4 Table**; down-regulated genes (see **S2F Fig**) are listed in **S5 Table**). A total of 441 genes were selectively up-regulated in WT, 60 were selectively up-regulated in *Krt17* null, and 268 were up-regulated in both genotypes (**Fig 2H**). To investigate which signaling pathways are affected by *Krt17* expression in TPA-induced skin in vivo, the 441 genes up-regulated in WT only and 268 genes up-regulated in both WT and *Krt17* null skin were separately analyzed using the Panther overrepresentation test and Reactome pathway tool [33,34] (note: too few genes (*n* = 60) were up-regulated in *Krt17* null skin to yield significant pathway findings). Several pathways are significantly overrepresented (FDR-adjusted *p*-value <0.05) in WT skin and in both WT and *Krt17* null skin (**Figs 2I and S2G**). The “innate immune system” and “neutrophil degranulation” were the pathways most significantly enriched in a K17-dependent fashion; moreover, virtually all other pathways showing significant enrichment relate to neutrophil or innate immune processes (**Figs 2I** and **S2G**). Though there was overlap in pathway significance, there is a significant difference in individual genes showing up-regulation in the K17-dependent versus K17-independent groupings (**S4 Table**). Taken together, this unbiased transcriptomic screen provides independent support for a significant role for K17 in regulating the skin’s response to stress, and the innate immune/neutrophil response in particular. Further insight gained from this RNAseq data set is reported below.

### K17 regulates neutrophil recruitment by promoting release of CXCR2 and CXCR3 ligands

**Fig 3:**
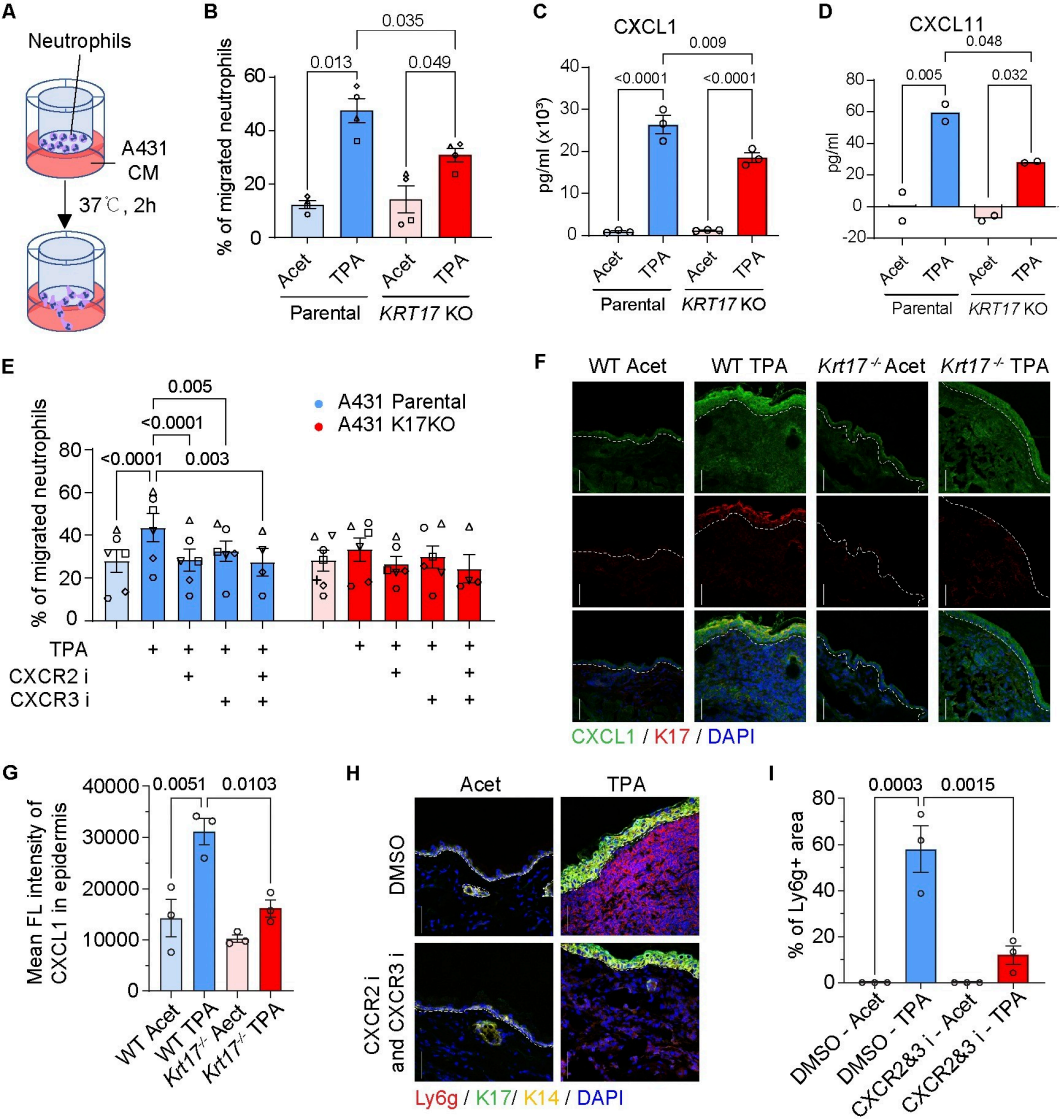
K17 promotes the secretion of neutrophil CXCR2 and CXCR3 ligands. **(A)** Schematic of neutrophil transwell migration assay. **(B)** Migration of human primary neutrophils towards CM from A431 keratinocyte cultures. Individual symbols depict data using neutrophils from different donors (*n* = 4). Data are shown as mean ± SEM. One-way ANOVA. **(C, D)** ELISA measurements of selected chemokine levels in A431 CM (pg/ml). *N* = 3 measurements for CXCL1 (2 technical replicates each), *n* = 2 measurements for CXCL11 (4 technical replicates each). Data are shown as mean ± SEM. One-way ANOVA. **(E)** Migration of human primary neutrophils towards A431 CM in the presence of CXCR2 antagonist and/or CXCR3 antagonist. Individual symbols depict data using neutrophils from different donors (*n* = 4–6). Data are shown as mean ± SEM. Two-way ANOVA. **(F)** Sections of WT and *Krt17*^*-/-*^ mouse skin harvested 6 h after double TPA (24 h apart), immunostained for CXCL1 and K17. Nuclei were stained with DAPI. Dashed lines depict the dermo-epidermal interface. Scale bars: 50 μm. **(G)** Quantitation of the fluorescence signal for CXCL1 in epidermis from (F). *n* = 3 mice. Data are shown as mean ± SEM. One-way ANOVA. **(H)** Impact of pretreatment with CXCR2 and CXCR3 inhibitors 30 min prior to a second TPA Tx in WT mouse skin. Time interval between the 2 TPA Tx was 24 h. Sections were immunostained for Ly6g, K17, and K14. Nuclei were stained with DAPI. Scale bars: 50 μm. **(I)** Quantitation of neutrophil fluorescence signal (surface area measurements) from (H). *n* = 3 mice. Data are shown as mean ± SEM. One-way ANOVA. The source data used to derive the numerical values reported here can be found in S1 Data.

We next aimed to explore whether the keratinocyte-driven and K17-dependent neutrophil recruitment observed in mouse skin is also replicated in human. To this end, we first examined whether human keratinocyte-derived signals can trigger chemotaxis of human neutrophils in a K17-dependent manner. We utilized the A431 keratinocyte cell line, an established model derived from human epidermoid carcinoma, which expresses K17 at baseline in culture and for which we have a *KRT17* null variant [25] (**S3A Fig**). We tested for a neutrophil chemotactic response to conditioned medium (CM) collected from parental or *KRT17* null cells using transwell migration assays (**Fig 3A**). CM from TPA-treated parental cells markedly stimulated the chemotaxis of human neutrophils relative to CM from vehicle-treated cells (**Fig 3B**). By contrast, the chemotactic response to CM obtained from TPA-treated *KRT17* null cells was significantly reduced (**Fig 3B**). Serial dilution of CM from both cell types showed a dose-dependent neutrophil response, suggesting that it entails a receptor–ligand interaction (**S3B Fig**). Restoration of K17 expression by CMV-driven expression of an EGFP-WTK17 fusion in TPA-Tx *KRT17* null A431 cells rescued neutrophil chemotaxis (**S3C Fig**). Together, these findings show that CM derived from TPA-stressed A431 keratinocytes induces neutrophil directional migration owing to the presence of specific chemotactic factor(s) that are produced and/or secreted in a K17-dependent manner.

ELISA assays were performed to compare the composition of CM from TPA-Tx parental and *KRT17* null A431 cells (**S3 Table**). We prioritized analysis of secreted signals known to impact neutrophil migration, e.g., CXCL1 and CXCL2 [35], which act through CXCR2, and CXCL9 and CXCL11 [36,37], which act through CXCR3. In addition to CXCR2, neutrophils under stress also express CXCR3 (see **S3D Fig** and refs [36,37]); besides, CXCL9 and CXCL11 expression shows a dependence upon K17 in stressed human and mouse keratinocytes [25,29]. We found that CXCL1, CXCL2, CXCL9, and CXCL11 chemokines occur at higher levels in CM isolated from TPA-Tx parental relative to *KRT17* null A431 cells (these differences reached statistical significance for CXCL1 and CXCL11; see **Figs 3C, 3D**, **S3E, and S3F**). Immunofluorescence staining for CXCL1, the most predominant chemokine produced by TPA-treated A431 cells ex vivo (**S3 Table**), revealed a marked increase in this chemokine in WT epidermis following dual TPA Tx compared to vehicle control, especially in K17-expressing suprabasal keratinocytes (**Fig 3F and 3G**). The observed increase in CXCL1 staining in the dermis of TPA-treated skin compared to control skin may result from extracellular CXCL1 released from epidermal keratinocytes and/or from dermal cells including fibroblasts, macrophages, and endothelial cells, which likely up-regulate CXCL1 in response to cytokines released by TPA-stressed keratinocytes. Importantly, CXCL1 immunostaining is significantly weaker in *Krt17* null epidermis compared to WT epidermis (**Fig 3F and 3G**), providing additional evidence that the up-regulation of CXCL1 is K17-dependent. Pretreatment of parental A431 cells with antagonists for CXCR2 (AZD5069) [38] or CXCR3 (AMG487) [39], or both, prior to TPA exposure significantly reduces CM-induced neutrophil migration (**Fig 3E**; antagonist specificity was confirmed using fMLF; **S3H Fig**). To verify whether CXCR2 and CXCR3 ligands are essential in vivo for driving neutrophil recruitment to inflamed mouse skin, we pretreated mouse skin with these receptor antagonists before the second TPA stimulation. Again, a significant decrease in neutrophil infiltration is observed after blocking these 2 chemokine receptors (**Fig 3H and 3I**). Collectively, these results indicate a role for CXCR2 and CXCR3 ligands and their receptors in mediating the keratinocyte- and K17-dependent recruitment of neutrophils, in both mouse skin in vivo and human keratinocyte cultures ex vivo.

### PKCα mediates the K17-dependent amplification of neutrophil influx in stressed skin

**Fig 4:**
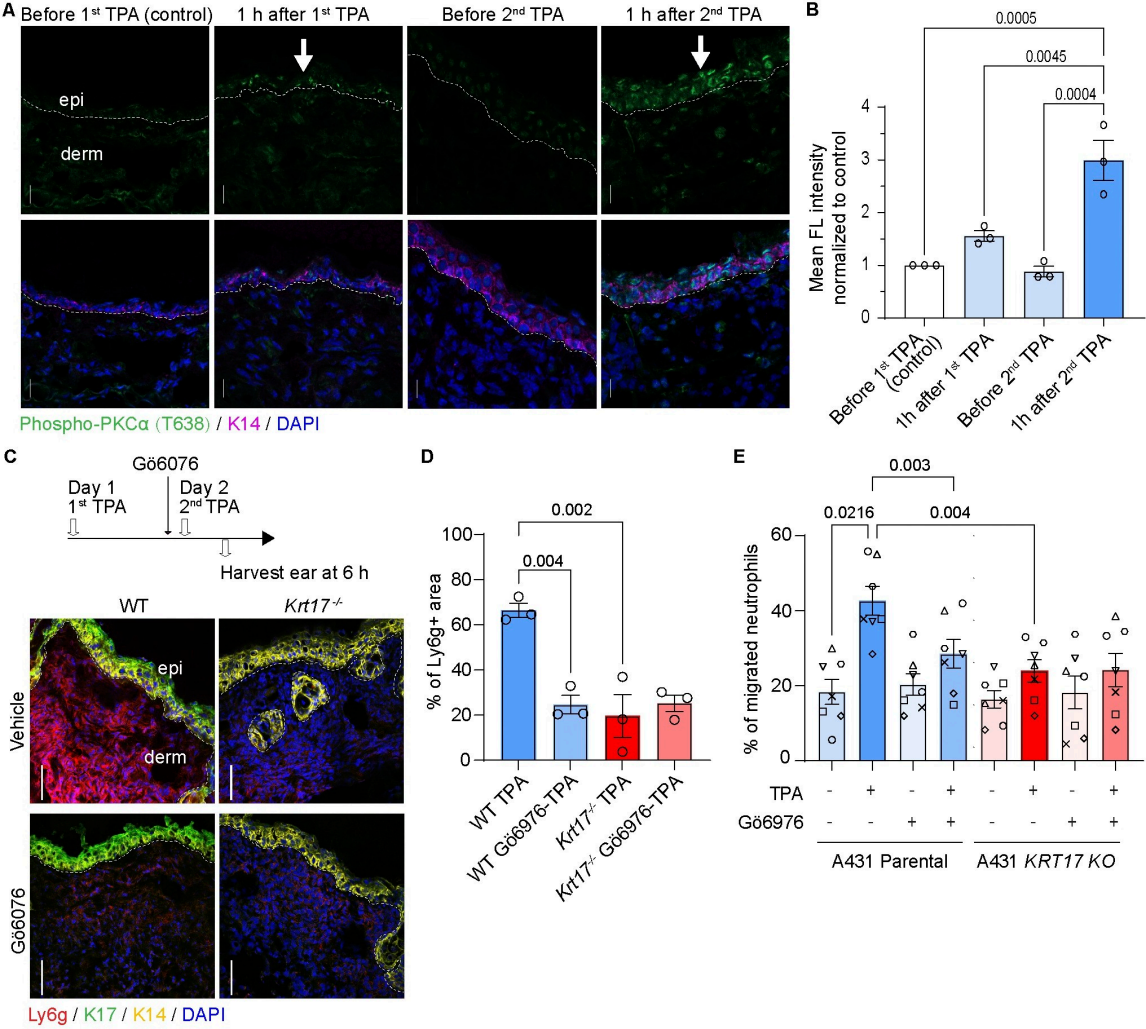
PKCα activity is crucial for K17-dependent neutrophil recruitment. **(A)** Sections of WT skin at baseline before Tx (control), 1 h after first TPA Tx, before second TPA Tx (24 h after the first TPA), and 1 h after second TPA were immunostained for phospho-PKCα (T638), K14, and nuclei (DAPI). Scale bars: 50 μm. epi, epidermis; derm, dermis. Dashed lines depict the dermo-epidermal interface. **(B)** Quantitation of the signal for phospho-PKCα levels in epidermis from (A). *n* = 3 mice. Data are shown as mean ± SEM. One-way ANOVA. **(C)** Impact of pretreatment with Gö6976 at 30 min prior to a second TPA Tx in WT and *Krt17*^*-/-*^ mouse skin. Time interval between 2 TPA Tx was 24 h. Sections were immuno-stained for K14, K17, Ly6g, and nuclei (DAPI). Scale bars: 50 μm. **(D)** Quantitation of neutrophil fluorescence signal (surface area measurements) from (C). *n* = 3 mice. Data are shown as mean ± SEM. One-way ANOVA. **(E)** Migration of human primary neutrophils towards CM from A431 cells treated with TPA and Gö6976. Individual symbols depict data using neutrophils from different donors (*n* = 7). Data are shown as mean ± SEM. One-way ANOVA. The source data used to derive the numerical values reported here can be found in S1 Data.

PKCα, the direct target of TPA, fulfills key roles in skin including the regulation of inflammatory responses and of desmosome cell-cell adhesion [40–42]. Previous studies showed that massive overexpression of PKCα, driven to unphysiologically high levels by the use of a strong keratin promoter in epidermis, leads to a severe neutrophilic inflammation in TPA-Tx skin [40,42]. We next assessed endogenous PKCα activity in WT mouse skin treated with TPA using an antibody against phospho-PKCα (Thr638), which reports on the active form of the kinase [43]. The signal for phospho-PKCα (Thr638) is increased 1 h after a single topical TPA Tx and has returned to baseline by 24 h (**Fig 4A and 4B**). Application of a second TPA Tx at that time (24 h after the first Tx) results in a markedly higher signal for phospho-PKCα 1 h later (**Fig 4A and 4B**). By contrast, total PKCα levels are unaffected by TPA Tx in WT mouse skin (**S4A Fig**). Next, we treated mouse ear skin with Gö6976, a pharmacological inhibitor of PKCα [44], prior to the second TPA Tx to test whether this kinase participates in TAR. Gö6976 Tx significantly curtailed TAR in WT skin but had no impact on the already low-level infiltration of neutrophils in *Krt17* null skin (**Fig 4C and 4D**). These findings suggest that PKCα and/or its downstream effectors play a key role in mediating TAR in skin tissue in vivo. We extended these in vivo findings by showing that pretreatment of A431 keratinocytes with Gö6976 prior to TPA stimulation significantly reduced the ability of their CM to stimulate neutrophil chemotaxis (**Fig 4E**). These findings show that endogenous PKCα activity mediates neutrophil infiltration in a K17-dependent manner in mouse skin and in human keratinocytes treated with TPA.

We next wondered whether PKCα signaling is involved in eliciting TAR after sequential TPA and UVB Tx (see **Fig 1**). PKCα activity levels are also significantly elevated in the epidermis of ear skin treated with TPA and UVB, 24 h apart (**S4B and S4C Fig**). Pretreatment with Gö6976 prior to UVB Tx in TPA-primed skin resulted in a significant reduction of neutrophil dermal infiltration (**S4D Fig**). This was confirmed by measurements of surface area of neutrophil infiltration in the dermis (**S4E Fig**) as well as of the dermal depth of the neutrophil infiltrate from the dermal-epidermal interface (**S4F Fig**). The latter index revealed that the epidermis-proximal pool of neutrophils was preferentially impacted by pretreatment with Gö6976 in the setting of the TPA-UVB Tx (see white asterisks in **S4D Fig**). Accordingly, TAR does not require the sequential use of a direct PKCα activator such as TPA and may represent a general property of keratinocytes in acutely stressed skin.

### K17 regulates the subcellular localization and activity of PKCα in stressed keratinocytes

**Fig 5:**
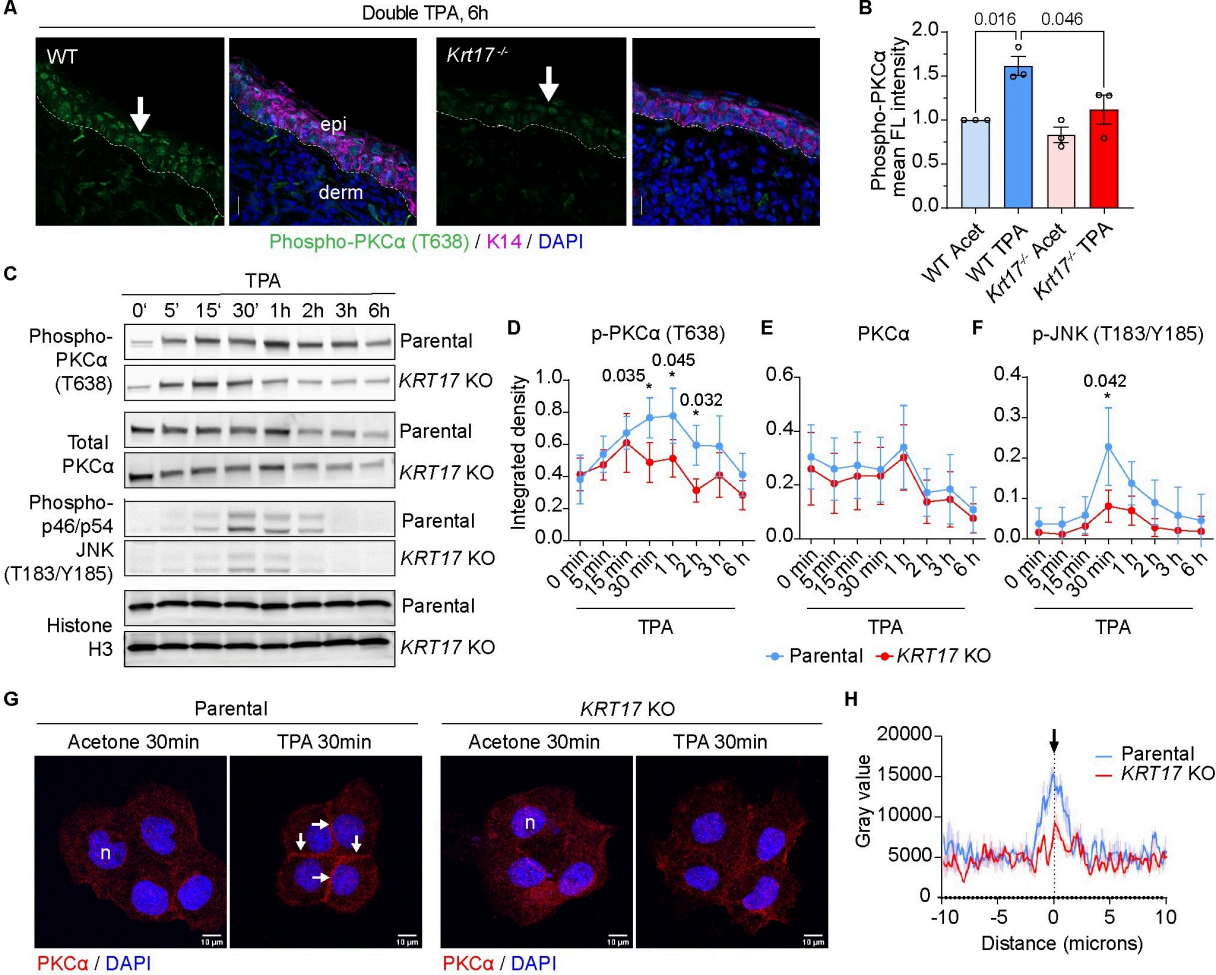
Subcellular localization and activity of PKCα are K17-dependent in A431 cells. **(A)** Sections of WT and *Krt17*^*-/-*^ mouse skin 6 h after double TPA Tx, 24 h apart, were immuno-stained for phospho-PKCα (T638), K14, and nuclei (DAPI). Scale bars: 50 μm. epi, epidermis; derm, dermis. Dashed lines depict the dermo-epidermal interface. **(B)** Quantitation of the phospho-PKCα signal from micrographs as shown in (A). *n* = 3 mice. Data reported as mean ± SEM. One-way ANOVA. **(C)** Western blot analysis of whole cell protein lysates at various times after TPA Tx (0–6 h; see top of blots). **(D–F**) Western blot quantitation of phospho-PKCα (T638), PKCα, and phospho-JNK (T183/Y185) normalized to histone H3, *n* = 3. Data reported as mean ± SEM. Two-way ANOVA. **(G)** Immunostaining for PKCα and nuclei (DAPI) in parental and *KRT17* KO A431 cells 30 min after vehicle or TPA Tx. Scale bars: 10 μm. Arrows point to PKCα staining at cell–cell interface after TPA Tx. **(H)** Quantitation of PKCα fluorescence intensity at cell–cell interface. *n* = 16. Data reported as mean ± SEM. Acet, acetone; n, nucleus. The source data used to derive the numerical values reported here can be found in S1 Data.

To investigate whether induced K17 participates in regulating PKCα in stressed keratinocytes, we compared the response of epidermis from WT and *Krt17* null mice to dual TPA Tx, 24 h apart. Immunostaining of tissue sections for phospho-PKCα evidenced a weak signal in WT and *Krt17* null epidermis at baseline (see **Fig 4A and 4B**). After dual TPA Tx, the signal for phospho-PKCα is primarily concentrated in the epidermis and is significantly stronger in WT compared to *Krt17* null skin (**Fig 5A and 5B**). By contrast, PKCα protein itself occurs at similar levels in WT and *Krt17* null epidermis after dual TPA Tx, based on immunostaining (**S4A Fig**). These data suggest that K17 may act as an amplifier of PKCα activity in stressed skin keratinocytes in vivo.

We took advantage of A431 keratinocytes, in culture, to continue probing the role of K17 in regulating PKCα activity. Time-course analyses of the activation of PKCα (via phosphorylation), using western immunoblotting, suggest similar kinetics in parental and *KRT17* null A431 cells during the first 15 min after TPA addition (**Fig 5C and 5D**). In parental A431 keratinocytes, phospho-PKCα levels continue to increase until 1 h after TPA, then gradually decrease and have returned to baseline by 6 h. By comparison, such a sustained increase in phospho-PKCα does not occur in *KRT17* null A431 cells, which show lower levels of phospho-PKCα at 30 min, 1 h, and 2 h after TPA addition. As was the case in mouse skin, total PKCα protein levels are indistinguishable across the same time frame in parental versus *KRT17* null A431 cells (**Fig 5C and 5E**). In further support of a difference between genotypes, phosphorylation of JNK, a direct target of classic PKC [45], also occurs at reduced levels in TPA-Tx *KRT17* null relative to parental A431 keratinocytes (**Fig 5C and 5F**). Finally, using immunostaining, we find that translocation of PKCα to the cell membrane, a hallmark of its activation [46], occurs in TPA-treated parental A431 cells but significantly less so in *KRT17* null cells (**Fig 5G and 5H**). Taken together, these findings demonstrate a role for K17 in sustaining PKCα activity after exposure of keratinocytes to stress.

PKCα regulates cell–cell adhesion in epidermal keratinocytes by phosphorylating desmoplakin, leading to detachment of keratin IFs and destabilization of desmosomes [41]. Accordingly, we examined the status of desmosomes in TPA-Tx A431 cells in culture. Parental and *KRT17* null cells subjected to acetone vehicle Tx show indistinguishable desmoplakin and E-cadherin staining which, as expected, is concentrated at cell–cell junctions (**S5A–S5C Fig**). No significant loss of desmoplakin staining at cell–cell junctions occurs at 30 min and 1 h after TPA Tx in parental A431 cells (**S5A–S5C Fig**). In contrast, desmoplakin staining is reduced at cell–cell interface at 30 min and at 1 h after TPA Tx of *KRT17* null A431 cells (**S5A Fig, arrows with asterisks)**, with the mean fluorescent intensity of desmoplakin staining at the cell boundary significantly lower at both time points compared to parental cells **(S5B and S5C Fig**). The latter occurs despite attenuated PKCα activity in *KRT17* null cells (see **Fig 5**), suggesting that desmosomes are impacted by PKCα-independent mechanisms in the absence of K17 [47].

### K17 interacts with the essential PKCα scaffolding protein RACK1

**Fig 6:**
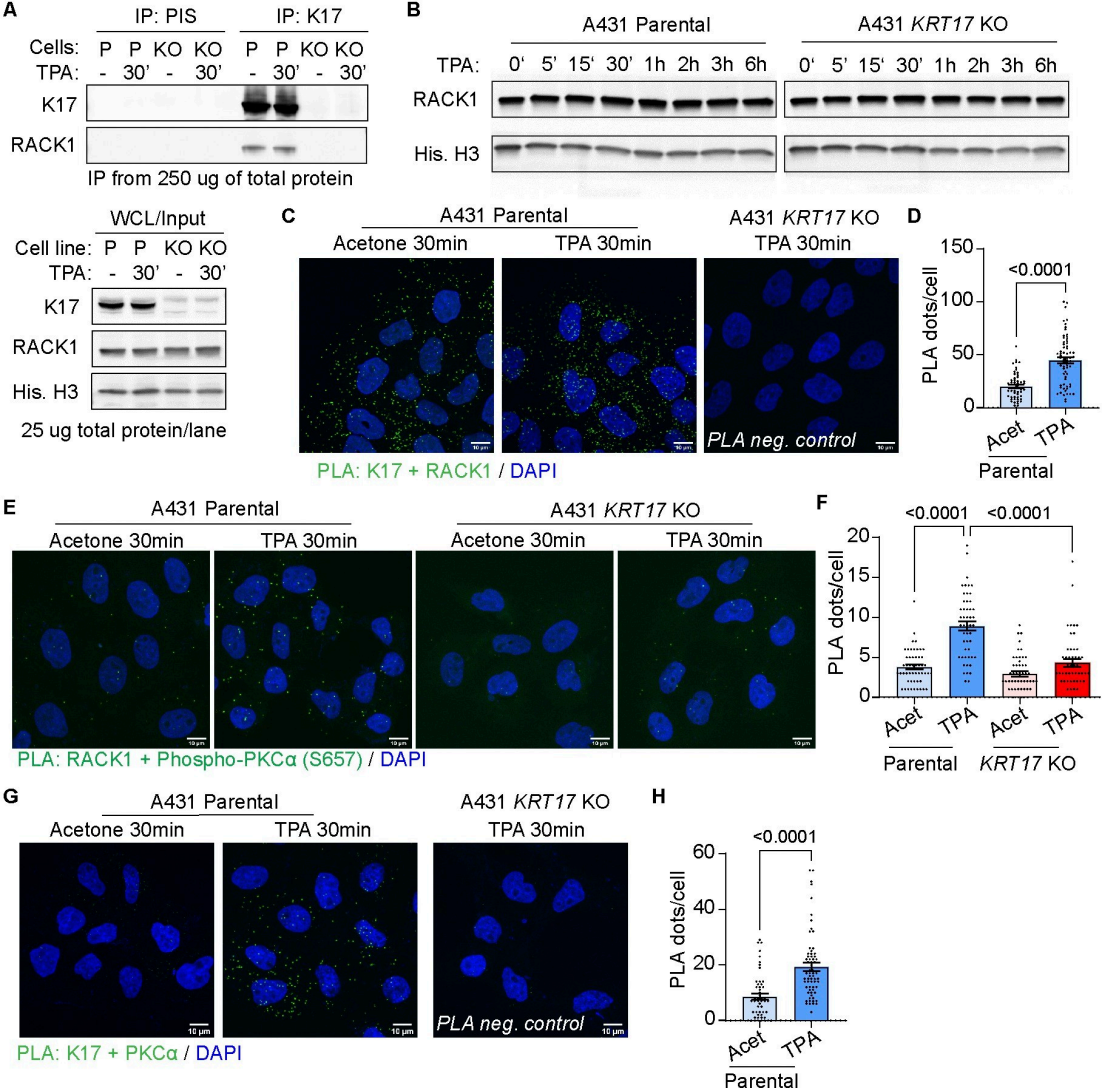
K17 interacts with the PKCα scaffolding protein RACK1. **(A)** RACK1 occurs in K17 immunoprecipitates (IPs) from detergent-soluble protein extracts prepared from parental and *KRT17* null A431 cells. Rabbit preimmune serum (PIS) was used as IgG control for IP, and input protein content is reported in bottom gel. **(B)** Western blot analysis of RACK1 level in parental and *KRT17* null A431 cells before and after TPA Tx. **(C–H)** PLA assays were performed for (C) K17 and RACK1, (E) RACK1 and phosphor-PKCα (S657) ([96]), and (G) K17 and PKCα in A431 keratinocytes treated with vehicle or TPA for 30 min. Maximum intensity projection (MIP) images are shown. Scale bars: 10 μm. In (**D**), (**F**), and (**H**), PLA signals were quantified as the number of PLA dots per cell. From left to right: *n* = 57 and 66 cells in (D), *n* = 51, 50, 50, 49 cells in F, *n* = 51, 67 cells in (H). Data are shown as mean ± SEM. Unpaired *t* tests were performed in (D) and (H) and one-way ANOVA was done in (F). Acet, acetone. The source data used to derive the numerical values reported here can be found in S1 Data.

The scaffolding protein RACK1 binds classic PKC isozymes and is essential for their activation and enhanced partitioning to the outer cell membrane [48,49]. Kroger and colleagues showed that RACK1 physically interacts with K5, a type II keratin, in mouse keratinocytes [41] and Yang and colleagues reported the presence of RACK1 in K17 immunoprecipitates from human HaCaT cells [50]. Our analyses show that RACK1 co-immunoprecipates with K17 in protein lysates from A431 keratinocytes, whether treated with TPA or not (**Fig 6A**), and that steady-state levels for RACK1 protein are similar in parental and *KRT17* null A431 cells (**Fig 6B**). Use of PLA assays shows that proximity of RACK1 and K17 is robust in parental A431 cells at baseline (acetone control), and is enhanced approximately 2.5-fold (*P* < 0.02) at 30 min after TPA Tx (**Fig 6C and 6D**). PLA assays also show enhanced proximity between RACK1 and phospho-PKCα (*P* < 0.0001; **Fig 6E and 6F**), and between K17 and PKCα (*P* < 0.0001; **Fig 6G and 6H**) at 30 min after TPA Tx in parental A431 cells. Both are significantly reduced, however, in *KRT17* null A431 cells. We conclude that K17 is bound to RACK1 in cultures of A431 keratinocytes at baseline but their interaction markedly changes early after TPA Tx, coinciding with enhanced proximity between RACK1 and active PKCα.

Keratins behave as insoluble proteins in aqueous salt solutions but are readily solubilized in buffers containing a high content of strong denaturing agents such as urea [51]. Besides, the solubility of keratin proteins is naturally affected by PTMs, the most studied of which is phosphorylation [52]. We next applied a Triton X-100-based fractionation protocol [53] to monitor the partitioning of K17, RACK1, and PKCα proteins in parental A431 keratinocytes after Tx with TPA. K17 is most abundant in the detergent-insoluble fraction, as expected, but also occurs in low salt buffer and detergent-soluble fractions (**S5D Fig**). At 1 h after TPA Tx the Triton-soluble pool of K17 is slightly increased (**S5D Fig**), likely reflecting a remodeling of K17-containing filaments [54]. Significant amounts of RACK1 and PKCα are extracted by the low salt buffer, as expected, while RACK1 but not PKCα also occurs in the Triton-soluble pool (**S5D Fig**). Interestingly, we observed that a small amount of RACK1 and PKCα co-partitions with K17 to the keratin-rich detergent-insoluble pool (**S5D Fig**). These findings are consistent with the PLA data but do not conclusively inform on the pool of K17 that interacts with RACK1 and regulates PKCα.

### The N-terminal head domain of K17 regulates RACK1 and PKCα activity

**Fig 7:**
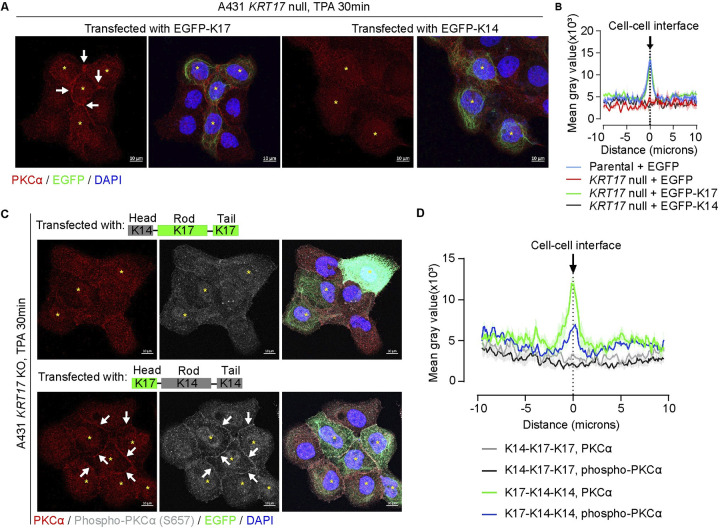
The N-terminal head domain of K17 is crucial for PKCα regulation. **(A)** Rescue assays involving expression of EGFP-WTK17 and EGFP-WTK14 fusion proteins in *KRT17* KO A431 cells. Cells were treated with TPA for 30 min and were stained for PKCα and nuclei (DAPI). Examples of transfected cells are identified by asterisks. Scale bars: 10 μm. **(B)** Quantitation of PKCα fluorescence intensity across cell–cell interfaces of adjoining transfected cells shown in (A). *n* = 12 cell for parental + EGFP (control); *n* = 16 for *KRT17* KO + EGFP (control); *n* = 22 for *KRT17* KO + K17-EGFP; *n* = 14 for *KRT17* KO + K14-EGFP. Data are shown as mean ± SEM. **(C)** Expression of EGFP-K17head-K14rod-K14tail and EGFP-K14head-K17rod-K17tail chimeric fusion cDNAs in *KRT17* KO A431 cells. Cells were treated with TPA for 30 min and immunostained for PKCα and phospho-PKCα (S657), and for nuclei (DAPI). Scale bars: 10 μm. **(D)** Quantitation of PKCα and phospho-PKCα (S657) fluorescence intensity across cell–cell interfaces in adjoining transfected cells as shown in (C). *n* = 20 for K17-K14-K14 group, and *n* = 16 for K14-K17-K17 group. Data reported as mean ± SEM. In (**A**) and (**C**), arrows depict staining for PKCα and phosphor-PKCα at the plasma membrane in adjoining cells expressing EGFP-WTK17- or EGFP-K17-K14-K14. The source data used to derive the numerical values reported here can be found in S1 Data.

In an effort to map *cis*-acting determinants in K17 that mediate regulation of PKCα activity, we exploited the observation that expression of a EGFP-WTK17 fusion [25] in *KRT17* null A431 cells rescues the TPA-induced recruitment of PKCα to the plasma membrane (PM) in TPA-treated transfected cells (**Fig 7A and 7B**, note that proximal A431 null cells not expressing EGFP-WTK17 failed to show PKCα recruitment to the PM in this setting). Expression of EGFP-WTK14, which is highly related to K17 in primary structure [12] and constitutively expressed in epidermis, led to filament formation but did not rescue the PM localization of PKCα in TPA-Tx *KRT17* null A431 cells (**Fig 7A and 7B**). Next, we constructed K14-K17 chimeras and tested them in this rescue assay to map the domain(s) within K17 that confers PKCα activation. Expression of an EGFP-K17head-K14rod-K14tail chimera, but not an EGFP-K14head-K17rod-K17tail chimera, succeeded in rescuing PM localization in transfected and TPA-Tx *KRT17* null A431 cells (**Figs 7C and 7D**), suggesting that the N-terminal head domain is key to K17’s ability to regulate PKCα.

**Fig 8:**
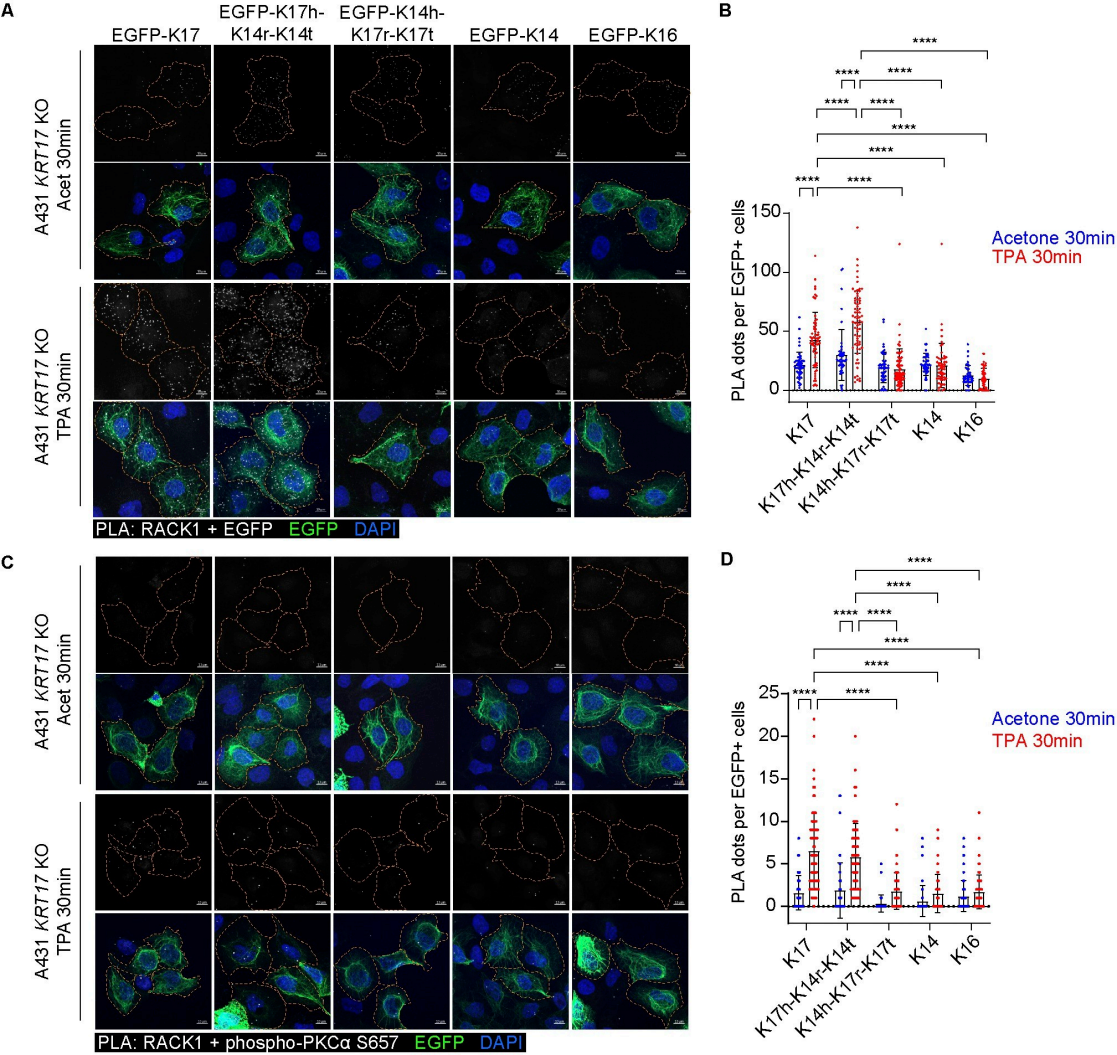
The N-terminal head domain of K17 plays a role in regulating RACK1. **(A, C)** Rescue assays involving expression of EGFP-WTK17, EGFP-K17head-K14rod-K14tail, EGFP-K14head-K17rod-K17tail, EGFP-WTK14, and EGFP-WTK16 fusion proteins in *KRT17* KO A431 cells. Cells were treated with TPA for 30 min and PLA assays were performed for performed for (A) GFP and RACK1, and (C) RACK1 and phospho-PKCα (S657). Maximum intensity projection (MIP) images are shown. Orange dashed lines depict the boundary of EGFP+ cell that are intact in the field of view. Scale bars: 10 μm. Acet, acetone. **(B**, **D)** PLA signals in cells that express EGFP-tagged keratin were quantified as the number of PLA dots per cell. From left to right: *n* = 54, 62, 46, 71, 48, 72, 43, 55, 40, 55 cells in (B), *n* = 47, 77, 44, 68, 40, 86, 45, 80, 53, 70 cells in (D). Data are shown as mean ± SEM. Two-way ANOVA. **** *P* < 0.0001. The source data used to derive the numerical values reported here can be found in S1 Data.

We next investigated whether the head domain of K17 plays a role in influencing the interaction between keratin and RACK1, as well as the interaction between RACK1 and phospho-PKCα. Using PLA in *KRT17* null A431 cells transfected with various constructs, we observed a significant enhancement in the proximity between EGFP-tagged keratins and RACK1, as well as between RACK1 and phospho-PKCα, in cells transfected with EGFP-K17 or EGFP-K17head-K14rod-K14tail chimera following TPA Tx compared to acetone control Tx (**Fig 8A–8D**). This significantly enhanced proximity does not occur in cells expressing EGFP-K14head-K17rod-K17tail chimera, or those expressing EGFP-K14 or EGFP-K16 (**Fig 8A–8D**), 2 keratins that share a highest amino acid identity with K17 [12]. We conclude from this that the head domain of K17 is crucial for the TPA-induced modulation in the RACK1-K17 interaction, which may subsequently impact the interaction between RACK1 and active PKCα.

### Relevance to chronic human inflammatory skin diseases

**Fig 9:**
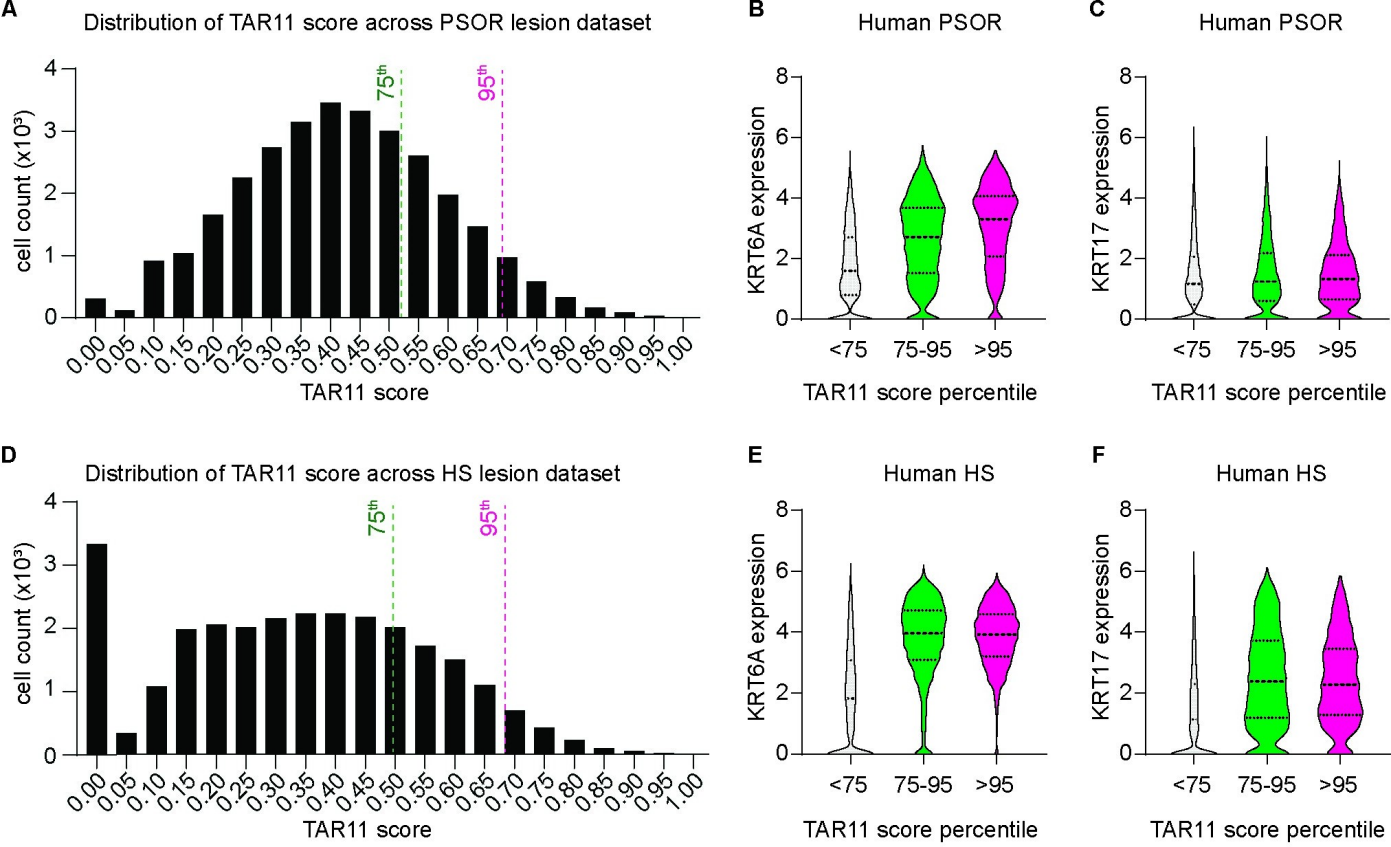
A TAR transcriptional signature occurs in stressed keratinocytes from human inflammatory skin diseases. A composite score, TAR11, was devised based on integrated analyses of transcriptomics datasets from TPA-Tx WT mouse skin and from human psoriasis (see main text) and used to query single-cell RNA sequencing datasets from (A–C) psoriasis (PSOR) and (D–F) hidradenitis suppurativa (HS) in human patients. **(A, D)** Distribution of TAR11 scores in all individual cells from (A) PSOR and (D) HS. **(B, C, E, F)** Violin plots reporting on expression levels for (B, E) *KRT6A* and (C, F) *KRT17* in cells showing high, medium, and low TAR11 scores. Cut-offs were arbitrarily set at the 95th percentile for a high TAR11 score and at the 75th percentile for a medium TAR11 score based on the distributions shown in A and D. The source data used to derive the numerical values reported here can be found in S1 Data.

We next explored whether one can uncover a transcriptional signature consistent with TAR, as seen in mice, in human skin disorders with a known neutrophil involvement (see below). We focused on up-regulated genes (>8-fold; adj. *P* < 0.01) in WT but not *Krt17* null skin at 6 h after dual TPA Tx, 24 h apart. Of the 441 genes in this group (see **Fig 2H**), 234 have known human homologs and 209 occur in a well-curated PSOR single-cell (sc) RNAseq data set [55] (**S6A** Fig and **S6 Table**). Analysis of these 209 genes using the Panther statistical overrepresentation test [33,34] indicated an enrichment for genes involved in neutrophil function and regulation, e.g., Dectin-2 family [56], GPVI proteins [57], and cytokine and chemokine production (**S7 Table**). Most of these 209 genes show preferred expression in clusters typified by myeloid lineage markers (LYZ), CD16, and additional neutrophil markers (“Myeloid-lineage” cluster) (**S6B Fig**). This said, a subset of 11 genes shows higher expression in skin keratinocytes over myeloid-lineage cells—these are *ANGPTL4*, *IFITM1*, *IFI16*, *PHLDA2*, *KLK1*, *PDPN*, *AKR1B10*, *ACAT2*, *TUBB6*, *OAS1*, *HRH2* (**S6C Fig**). Remarkably, 6 of these genes, *AKR1B10* [58], *ANGPTL4* [59], *OAS1* [60], *HRH2* [61], *KLK1* [62], and *PDPN* [63] have known roles in regulating PKCα or other classic PKCs, or at steps downstream from their activation. Using these 11 genes, we devised a composite expression score (TAR11 score) and used it to query PSOR [55], AD [64], and HS [4] scRNAseq data sets (see Methods and ref. [10]). The distribution of TAR11 scores in single cells of lesional PSOR (**Fig 9A**) and HS skin (**Fig 9D**) is quasi-normal. Cells with the highest TAR11 scores (cut-off set at either the 95th and 75th percentiles) also show the highest levels of reads for inflammatory, immune response, and stress response genes including the highest levels of *KRT6A* and *KRT17* mRNAs (**Fig 9B, 9C, 9E, and 9F**). A similar outcome is observed in a scRNAseq data set from AD patients (**S6D and S6E Fig**).

To determine whether these 11 genes respond to a single acute stimulation or are exclusively up-regulated by repeated stimulation, we compared fold changes observed in WT mice in our data set (6 h after dual TPA Tx) with those occurring in WT mice that received a single topical TPA Tx (4 h time point) [65]. The results indicate that *Acat2*, *Ifi209*, and *Hrh2* are significantly up-regulated only following repeated stimulation (**S8 Table**). *Ifitm1*, *Akr1b8*, *Oas1a*, and *Pdpn* are up-regulated following a single stimulation—this said, the extent of their up-regulation is notably greater after repeated stimulation (**S8 Table**). This suggests that these 7 genes are associated with the response of skin to repeated inflammatory stress. Immunofluorescence staining confirmed that the protein levels of HRH2 and OAS1 are significantly elevated in the WT epidermis after dual TPA Tx, but not in *Krt17* null epidermis (**S6G–S6J Fig**).

The outcome of these computational analyses is significant in 3 ways. They show that (i) the transcriptional signature of WT mouse ear skin when TAR is manifested (i.e., at 6 h after 2 TPA Tx, 24 h apart) shows a strong neutrophil character, as expected; (ii) several of the genes up-regulated in TAR-exhibiting mouse skin are expressed in human inflammatory skin disorders and are involved in regulating PKC activity; and (iii) a transcriptional signature inferred from TAR as it occurs in mouse skin is shared with keratinocytes exhibiting high expression of stress keratins, including *KRT17*, in human skin disorders.

## Discussion

We show here that the cytoplasmic pool of K17 induced by stress in epidermal keratinocytes amplifies neutrophil recruitment in the superficial and deeper dermis upon additional stress exposures. This property represents a short-lived, transient adaptation that entails the K17-dependent regulation of PKCα activation and release of classic neutrophil chemokines. Analysis of transcriptomic data suggests that the observations made in acutely stressed mouse skin may be relevant to human chronic skin inflammatory diseases such as PSOR, AD, and HS. These findings and their implications significantly advance our understanding of the relevance and roles of stress keratins, PKCα, and their partners in complex epithelia such as skin [10].

Classic examples of adaptation to stress include the photoprotective pigmentation that follows UV radiation in skin [66] and adaptive and innate immune cell responses [67]. Recently, a new mechanism has been described whereby skin epithelial stem cells develop a stable epigenetic memory of past exposures to stress [68–70]. This memory persists for >6 months after an initial insult, entails a Stat3- and Fos/Jun-dependent open chromatin state in epithelial stem cells, and enhances the skin’s sensitivity to subsequent stimuli [68,69]. The TAR described here is distinct as it is driven by epidermal keratinocytes, is short-lived (<48 h), and regulated in part by K17-dependent PKCα signaling at the cell periphery. TAR may serve the purpose of transiently protecting skin against additional stress exposures during a period of enhanced keratinocyte vulnerability. Others have shown that AP-1 [71] and Stat3 [72] regulate *KRT17* transcription, and that the nuclear pool of K17 protein modulates Stat3 function in keratinocytes of inflamed skin [26]. Conceivably, stresses that induce a short-term TAR in differentiating keratinocytes of the epidermis may trigger a longer-lived epigenetic memory in epithelial stem cells of the tissue.

In the course of this study, we explored the ability of various types of acute stresses (e.g., TPA, UVB, UVA, Imiquimod) to elicit a TAR-like response in mouse ear skin. These efforts showed that TAR manifests only when TPA is applied as the initial insult. The latter correlates with the observation that, of the stressors tested, only TPA results in a robust and prolonged (up to 48 h) induction of K17 in treated epidermis within hours after a single exposure. TAR does not occur when the second stress (TPA or UVB) is administered at 48 h, instead of 24 h, after the initial TPA priming event. While necessary, therefore, K17 is not sufficient to mediate the early phase of TAR. Transient factors present 24 h but not 48 h after a priming event may be required for TAR to develop in full. A posttranslational modification (PTM) on K17, for example, could be such a factor. PTMs can act in *cis* or *trans* to affect protein structure and/or interaction with other proteins [52]. Coincidentally the N-terminal head domain of K17, which is required for TPA-induced PKCα activation, undergoes phosphorylation on several Ser/Thr residues (e.g., Thr9, Ser10, Ser13, Ser32, Ser44) in response to TPA Tx and other stimuli [21,22,73]. For example, phosphorylation of Ser44 in the head domain of K17 persists for only a few hours after a single TPA exposure [22], and fosters its interaction with 14-3-3σ, enabling the resulting complex to promote protein synthesis and cell growth in keratinocytes challenged to grow [21]. This PTM event is not relevant here, however, since we found that expression of a K17 Ser44Ala mutant [21] in *KRT17* null A431 keratinocytes rescues the PKCα phenotypes, just like WT K17 does. K17 is also subject to SUMOylation [74] and ubiquitination [50,73], so other PTMs (and other protein partners) could have a positive influence on TAR as well. Alternatively, it could be that a negative regulator of TAR is present or effective at 48 h beyond the initial, K17-inducing priming event in skin. Solving this enigma requires additional studies but is poised to yield novel clues as to how to mitigate TAR in the setting of human chronic inflammatory skin diseases.

We showed here that K17 promotes the membrane recruitment and activity of PKCα in epidermal keratinocytes subjected to inflammation-inducing stress, both in cell culture and in situ. This newly defined property involves *cis*-acting determinant(s) located in the N-terminal head domain of K17 [12] and correlates with K17’s ability to bind RACK1, a scaffolding protein essential for PKCα activity [48,49]. Consistent with a role in regulating skin inflammation, PKC has been shown to regulate several types of signaling events involved in the pathogenesis of inflammation, including nitric oxide biosynthesis [75], inflammatory cytokine [76] and superoxide production [77], and phospholipase A2 activation [78]—moreover, increased levels of diacylglycerol [79] and of PKCα [80] occur in psoriatic skin. While different in nature, ours in not the first documentation of a functional interaction between keratins and PKCα. Indeed, Kroger and colleagues [41] reported that K5-K14 filaments promote the genesis of calcium-induced, stable adhesions between keratinocytes by physically sequestering PKCα away from desmosomes, showcasing K5-K14 as negative regulator of PKCα activity. The data reported here point to K17 as a positive regulator of both the membrane association and activity of this kinase. Such a different outcome highlights the need for deeper studies of the mechanism and regulators of the keratin-RACK1-PKCα signaling axis in stressed epidermal keratinocytes.

More generally, our findings add evidence linking all subclasses of IFs to the regulation of cellular kinases with direct significance for several cellular processes. Thus K6, a type II keratin that is partially co-regulated with *KRT17*/K17 in stressed and diseased skin [10], directly binds Src, inhibiting its activity and promoting the coordination of cell migration and adhesion within keratinocyte sheets during wound re-epithelialization [81,82]. K8/K18, a prominent keratin pair in simple epithelia, binds to and stimulates the phosphorylation-dependent activation of Akt1, promoting hepatocyte survival after liver injury [83]. K10, a type I keratin expressed at an early stage in differentiating keratinocytes of epidermis, also binds Akt, leading to its sequestration and cell cycle arrest [84]. Vimentin, a type III IF, binds directly to CaMKII in smooth muscle cells and sequesters it to regulate contractility. Nestin, a type IV IF, binds directly to and sequesters Cdk5 to promote survival of neuronal progenitor cells [85] and also binds Plk1 to positively regulate its phosphorylation and smooth muscle contraction [86]. The K17-PKCα and, more generally, IF-kinase partnerships provide compelling examples of the growing role of IFs in regulating key signaling pathways in a biological context-dependent manner [10,17,20,82].

Comparative and integrative analysis of bulk RNAseq data collected from mouse skin under stress and single-cell RNAseq data collected from chronic inflammatory skin diseases suggest that the TAR mechanism newly revealed in this study is conserved in the human and possibly relevant to chronic inflammatory skin diseases. Remarkably, the TAR11 signature emanating from this comparative and integrative analysis includes 6 genes whose protein products have a known involvement in PKCα or classic PKC isoform regulation (see **S7 Table**). Future studies are needed to test and refine the “TAR11” signature and assess whether enhanced PKCα activity, in particular, plays a significant role in the genesis and/or maintenance of skin lesions in disorders such as PSOR, HS, AD, and others. Likewise, the role of additional cellular effectors of innate immune responses in skin, such as macrophages, Langerhans cells, and dendritic epidermal T-cells [87] in modulating TAR needs be examined. Meanwhile, the findings we report here deepen our appreciation of the paracrine and cell-autonomous pathways that shape the skin’s response to repeated stresses and may inform future research to devise new biomarkers or therapeutic targets for neutrophil-driven inflammatory skin diseases.

## Materials and methods

### Ethics statement

Heparinized whole blood from anonymous healthy human donors was obtained by venipuncture from the Platelet Pharmacology and Physiology Core at the University of Michigan (IRB#HUM00107120). Samples were deidentified and study authors did not have access to the HIPAA information.

### Mouse models and treatments

All mouse experiments involved 2- to 3-month-old male mice in the FVB background and were approved the Institutional Animal Use and Care Committee of University of Michigan Medical School. The *Krt17*^−/−^ [31] and *Krt17*^*ΔNLS/ΔNLS*^ [24] mouse strains were previously described. Mice were treated with 20 μl of 0.2 mg/ml of TPA (Cell Signaling Technology #4174S), topically on the dorsal side of ear skin, with the other ear treated with acetone vehicle control. For acute UVB treatment, mice were anesthetized with isoflurane and put in a dark cabinet equipped with 4 UVB lamps (305 to 312 nm) (Daavlin Distributing Company). The dorsal side of ear skin was exposed to 400 mJ/cm^2^ of UVB radiation [88]. For topical treatment of Gö6976, 100 μm Go6976 (Cell Signaling Technology #12060S) dissolved in 10 μl acetone was applied to the dorsal side of mouse ear 30 min before the second TPA treatment. For topical treatments involving CXCR2 and CXCR3 inhibitors, 100 μm of AZD5069 (CXCR2 inhibitor; Cayman Chemical Company #28297) and 100 μm of AMG487 (CXCR3 inhibitor; Tocris #4487) were mixed together in 20 μl of DMSO and applied to the dorsal side of mouse ear 30 min before the second TPA treatment.

### Tissue collection and cryosectioning

Mice receiving treatments were euthanized and ear skin tissue was collected at specific time points as indicated. Ear samples were embedded in −40°C optimal cutting temperature compound (O.C.T, Sakura Finetek USA, #4583). Cryosectioning was performed at −20°C using a CRYOSTAR NX50 Cryostat (Thermo Scientific) and MX35 ultramicrotome blade (Epredia #3053835), and 5-μm thick cross-sections were cut and placed on positively charged microscope slides (VWR #48311–703) and stored at −40°C until further use.

### Cell lines and treatments

A431 cells were obtained from the American Type Culture Collection (ATCC). A *KRT17* null A431 variant was described [25]. Cells were cultured in DMEM medium (Gibco #11995–065) supplemented with 10% FBS and 0.01% Penicillin-Streptomycin (Gibco #15140–122). Cells were treated with 20 nM TPA in acetone for the time indicated. For studies using Gö6976, 1 μm Gö6976 dissolved in acetone was added 30 min before TPA treatment.

### Indirect immunofluorescence and TUNEL staining

For frozen tissue sections, slides stored at −40°C were taken to room temperature, dried for 10 min, fixed with 4% paraformaldehyde (PFA) (Electron Microscopy Sciences #15710) for 10 min at room temperature, followed by 3 washes of 5 min with 1× PBS. A circle was drawn around each tissue section with a hydrophobic barrier pen (CALIBIOCHEM #402176). Tissue sections were blocked with blocking buffer (2% normal goat or donkey serum, 1% bovine serum albumin in 1× PBS) for 30 min at room temperature. For cultured cell samples, cells seeded on glass coverslips in 12-well or 24-well plates and cultured overnight at 37°C and 5% CO_2_ to let cells attached. After treatment, cells were fixed with 4% PFA for 10 min, permeabilized with 0.1% Triton X-100 for 5 min, then blocked with blocking buffer (5% normal goat or donkey serum in 1× PBS) for 1 h at room temperature. Unconjugated primary antibodies were diluted in blocking buffer and applied overnight at 4°C. On the second day, samples were washed and incubated with fluorophore-conjugated secondary antibodies for 1 h at room temperature in dark. Samples were then stained with 1 μg/ml of DAPI (Milipore Sigma #268298), washed, mounted with coverslips via FluoroSave reagent (EMD Millipore #345789), and dried overnight. The Click-iT Plus TUNEL Assay for in situ apoptosis detection with Alexa Fluor 488 (Invitrogen #C10617) was used as recommended by the manufacturer. Both tissue sections and cultured cell samples were imaged using a Zeiss LSM800 confocal microscope (Zeiss, Germany). The antibodies used are listed in **S1 Table**.

### Transient transfection of EGFP-tagged keratin constructs

The EGFP-K17WT and EGFP-K14WT constructs (pC3-EGFP vector backbone) have been described [24]. The K14-K17 chimeras were constructed by PCR-driven overlap extension [89] using the oligonucleotide primers listed in **S2 Table** with wild-type K17 and wild-type K14 cDNAs as templates. Constructs were transiently transfected into *KRT17* null A431 cells using the SF Cell Line 4D-Nucleofector X Kit (Lonza #V4XC-2032) and Lonza 4D-nucleofector X unit “A431 cell” program, and 0.5 μg of plasmid was used to transfect every 0.4 million cells. Transfected cells were plated on coverslips for immunofluorescence assays or in 6-well plates for conditioned media collection. The cells were allowed to rest for 48 h and then subjected to the desired outcome.

### Proximity ligation assays (PLA)

Cultured cells were fixed with 4% PFA solution for 15 min, permeabilized with 0.1% Triton X-100 solution for 10 min at room temperature, washed, blocked with 2.5% normal goat serum for 30 min at 37°C. Primary antibodies (**S1 Table**) diluted in blocking buffer were added and the preparations were incubated at 37°C for 1 h. The Duolink PLA Fluorescence kit (Millipore Sigma) was used according to the manufacturer’s instructions. Briefly, cells were incubated with PLA probes for 1 h at 37°C, with the ligation mixture for 30 min at 37°C, and with the amplification mixture for 100 min at 37°C. Cells were then stained with DAPI (1 μg/ml) for 10 min at room temperature and mounted on microscope slides. Images were captured using a Zeiss LSM800 confocal microscope.

### Western blotting and immunoprecipitation

Whole cell lysates were prepared in NP-40 lysis buffer [0.5% NP-40, 150 mM NaCl, 20 mM Tris (pH 7.5), EDTA 1 mM, 1× cOmplete protease inhibitor cocktail solution (Millipore #11836170001), 1× Pierce phosphatase inhibitor cocktail solution (Thermo Scientific #A32957), milliQ water]. Briefly, cultured cells in plates were washed once with cold 1× PBS and transferred to ice. Lysis buffer was added, and cells were scraped off from the plate and rotated at 4°C for 1 h. Supernatants were collected after centrifugation (12,000 × g, 20 min) and total protein levels were measured using Pierce BCA Protein Assay Kits (Thermo Scientific #23227). Proteins were denatured by boiling with Laemmli buffer (Bio-Rad #1610747) supplemented with 10% β-mercaptoethanol (β-ME) at 95°C for 10 min. SDS-PAGE electrophoresis was performed on 4% to 15% gradient gels (Bio-Rad #4561084) or 4% to 20% gradient gels (GenScript #M00656). Proteins on gels were transferred to nitrocellulose membranes (Bio-Rad #1620115) using a transblot turbo transfer system (Bio-Rad). Blots were blocked in 5% BSA in tris-buffered saline containing 0.1% Tween20 (TBST-T) for 1 h at room temperature and incubated overnight at 4°C with primary antibodies (cf. **S1 Table**) diluted in the blocking buffer. Secondary antibodies (cf. **S1 Table**) diluted in blocking buffer were applied for 1 h at room temperature. Blots were developed using SuperSignal West Pico PLUS chemiluminescent substrate (Thermo Scientific #34580) or ECL Select Western Blotting Detection Reagent (Cytiva #RPN2235), and imaged using a FluorChem Q system (ProteinSimple). For K17 immunoprecipitation (IP), Dynabeads Protein A Immunoprecipitation Kit (Invitrogen #10006D) was used, and 50 μl of beads was incubated with 2 μl of rabbit polyclonal K17 antibody at 4°C overnight. The same volume of rabbit preimmune serum was used as control. A431 cells (*KRT17* WT and *KRT17* KO) were lysed using NP-40 lysis buffer as described before. Whole cell lysates (250 μg of total protein) were incubated with antibody-beads complex at 4°C overnight. The eluted IP samples were denatured and subjected to western blotting.

### Harvesting conditioned medium (CM)

3 × 10^5^ A431 cells were seeded in each well of 6-well plates and cultured overnight in 2 ml of medium supplemented with full serum (10% FBS). On day 2, cells were treated with 20 nM TPA in full serum medium for 6 h. After rinsing (1× PBS), 2 ml serum-reduced medium (0.1% FBS) was added cells incubated for another 24 h. CM was collected using a 3 ml syringe (BD Syringe) and filtered through a 0.22 μm PVDF syringe filter (Millipore #SLGVM33RS), divided into aliquots and stored at −20°C until use. Measurements of selected cytokines and chemokines in CM were performed by RayBiotech and Immunology Core at University of Michigan.

### Isolation of human peripheral blood neutrophil

Blood from anonymous healthy human donors who had not taken aspirin for 7 days and NSAIDS for 48 h was obtained by venipuncture from the Platelet Pharmacology and Physiology Core at the University of Michigan. Neutrophils were isolated as described [90]. Briefly, whole blood was incubated with an equal volume of 3% dextran (Sigma #D1037) in 0.9% NaCl for 30 min at 37°C to facilitate red blood cell sedimentation. The upper layer (plasma) was collected and centrifuged at 400 × g for 5 min, and the cell pellet containing platelets, monocytes, lymphocytes, and neutrophils was collected and resuspended in 1× mHBSS (150 mM NaCl, 4 mM KCl, 1.2 mM MgCl_2_, 10 mg/ml glucose, and 20 mM HEPES). Histopaque-1077 solution (Sigma #10771) was carefully underlaid below the suspension and centrifuged at 400 × g for 20 min (ACC/DEC = 2/0) to separate peripheral blood mononuclear cells from neutrophils. Residual erythrocytes in the pellet were removed using ACK lysing buffer (Thermo Fisher #A1049201). Purified neutrophils were resuspended in 1× mHBSS and kept at 37°C until used. In our hands, this protocol yields >99% live neutrophils with >95% purity.

### Neutrophil transwell migration assays

Neutrophil transwell migration assays were performed in a transwell system consisting of a 24-well tissue culture plate and 3.0-μm pore size PET membrane inserts (Sterlitech Corporation #9323012). Plates and inserts were coated with 2% bovine serum albumin (BSA) for 1 h at 37°C to prevent neutrophil adhesion. Freshly isolated neutrophils (0.4 million cells in 100 μl of 1× mHBSS) were added into the upper chamber (insert) and 600 μl of CM or control medium (0.1% FBS) into the lower chamber (well of the plate). After a 2-h incubation at 37°C, the insert together with neutrophils remaining in the upper chamber were discarded. Cells in the bottom chamber were counted using a hemocytometer. The percentage of migrated cells was calculated by relating the cell number in the bottom chamber to total cells seeded in the upper chamber (0.4 million). Data was normalized by subtracting the percentage of migrated cells to control medium [91]. When testing the effect of inhibitors, freshly isolated neutrophils were pretreated with 1 μm of AZD5069 (CXCR2 inhibitor; Cayman Chemical Company #28297), 1 μm of AMG487 (CXCR3 inhibitor; Tocris #4487) for 30 min before the transwell migration experiments. A431-derived CM were supplemented with same concentration of inhibitors before used in migration assays. These inhibitors together with 1 μg/ml of TNFα neutralizing antibody Infliximab (Novus Biologicals #NBP2-52655) do not alter neutrophil migration in response to fMLF, a potent neutrophil chemoattractant [91], suggesting that neutrophils can respond normally to other chemotactic cues in the presence of the inhibitors (**S3H Fig**). The neutralizing antibody of TNFα was added due to its K17-dependent release (**S3G Fig and S3 Table**) and its suggested role in promoting neutrophil influx induced by antigen challenges in vivo [92].

### Quantitative real-time PCR analysis (qRT-PCR)

Neutrophils (10 million cells) were collected prior to treatment (t0) or after 2 h of rotation at 37°C for 2 h in mHBSS, reduced serum DMEM (0.1% FBS), CM of acetone-treated parental A431 cells, or CM of TPA-treated parental A431 cells. RNA isolation was then performed using Qiagen RNeasy mini kit (Qiagen #74104) following the manufacturer’s protocol. Then, total RNA was converted to complementary DNA (cDNA) using iScript cDNA Synthesis Kit (Bio-Rad **#1708891**). The cDNA obtained was subjected to qRT-PCR using the itaq Universal SYBR green kit (Bio-Rad, #1725122) and the CFX 96 Real-Time System (Bio-Rad). The PCR parameters for qRT-PCR screen were 95°C for 5 min, followed by 40 cycles of 95°C for 10 s and 60°C for 30 s. No cDNA template controls and a melt curve were included in every PCR run. The normalized expression value of the target gene was determined by first averaging the relative expression of the target gene for each cDNA sample (ΔCq = average Cqtarget gene—average Cq_reference gene_), and then normalizing the relative expression value of the experimental condition to the control condition (2^-(ΔCqExperimental- ΔCqControl)^). Primers used in qRT-PCR assays are listed in **S2 Table**.

### Mouse skin immune cell profiling (CyTOF)

The dorsal ears of mice were treated with TPA or acetone. Ear tissues were collected for isolation of immune cells. The tissues were rinsed in 10 ml of skin digestion medium (450 ml HBSS, 50 mlFBS, 1.1915 g HEPES, 0.9356 g EDTA, filtered through a 0.22 μm PVDF filter) for 30 min at 37°C. Then, the tissues were transferred to fresh 9 ml of skin digestion medium, cut into small pieces, and 1 ml of triple enzyme solution (1 mg/ml type I deoxyribonuclease, 1 mg/ml type V hyaluronidase, and 5 mg/ml collagenase, filtered through a 0.22 μm PVDF filter) was added and incubated for 30 min at 37°C. The solution was filtered through a 70 μm filter and centrifuged at 1,600 rpm for 5 min to form cell pellets. The cell pellet was resuspended with 1 ml of pre-warmed PBS and the cell number was counted. Cells were then centrifuged at 300 × g for 5 min and resuspended in 200 μl of PBS. Isolated cells were stained following Maxpar Cell Surface Staining with Fresh Fix protocol (Standard BioTools). Briefly, cell viability staining was performed by adding 50 μl of Cell-ID Cisplatin (Standard BioTools #201195) to the cell suspension. Cells were washed with Maxpar Cell Staining Buffer (Standard BioTools #201068), centrifuged at 300 × g for 5 min, and resuspended in 25 μl of staining buffer containing Fc block to incubate for 10 min at room temperature. Then, 50 μl of antibody mixture was added and incubated on ice for 1 h. Maxpar Cell Staining Buffer was added for washing and cells were collected after centrifugation. Cells were fixed with 1.6% PFA solution for 10 min at room temperature, centrifuged at 800 × g for 5 min. The cell pellet was resuspended in 1 ml of 125 nM Cell-ID Intercalator-Ir (Standard BioTools #201192A) made using Maxpar Fix and Perm Buffer (Standard BioTools #201067) and incubated overnight at 4°C. Conventional CyTOF was performed by the UMICH CyTOF Core. Final data were gated and analyzed using FlowJo.

### Bulk RNA sequencing

TPA or acetone was applied to the dorsal ears of *Krt17*^*-/-*^ and WT mice twice, 24 h apart (*n* = 3 mice for each group), and ears were collected at 6 h after the second treatment. RNA was isolated and purified using the Qiagen RNeasy mini kit (Qiagen #74104) and Qiagen RNase-Free DNase kit (Qiagen #79254) using the manufacturer’s instructions. RNA samples were transferred to the University of Michigan Advanced Genomics Core (AGC) for RNA quality control, RNA ribo-depletion library preparation, and next-generation sequencing (NovaSeq S4 300 cycle). Core supplied FASTQ files were mapped to the GRCm39.vm30 reference using STAR version 2.7.10a and the ENCODE standard options from the version documentation [93]. The reference matching GTF file was used with featureCounts from the Bioconductor R package Rsubread to create the counts matrix. The edgeR Bioconductor package was used to filter low expressing genes using the “filterByExpr” function, calculate log counts per million, and normalization factors using the weighted trimmed mean of M-values (TMM) method. The Limma Biocoductor package, with the precision weights, “voom” approach was used to perform linear models [94]. Differential gene expression was determined using custom thresholds as described for each analysis in the main text and in figure legends. The data is publicly available at the Gene Expression Omnibus (GSE245384).

### Analysis of human scRNAseq data sets

PSOR, HS, and AD data sets were previously published [4,55,64] and analyzed through standard Seurat SCTransform v2 pipeline [95]. In short, raw data was filtered to retain cells with [500<unique features<7,500] for PSOR data set or [500<unique features and <50,000 total counts] for HS and AD data set as well as a mitochondrial content <15%. Following the SCTransform “v2” normalization and variance stabilization, the top principal components (30 for PSOR, 15 for HS, 20 for AD) were used to construct a shared nearest neighbor (SNN) graph, and subsequently the Louvain algorithm was used to generate clusters (resolution parameter of 0.7 for PSOR, 0.6 for HS and AD). For composite scores, raw counts were library size normalized and log transformed. To identify gene homology (mouse to human), genes up-regulated in a K17-dependent manner post-TPA Tx (>8-fold; Adj. *P* < 0.01) were analyzed against the MGI Mouse-Vertebrate homology database, and 234 of the 441 genes were identified to have human homologs, totaling 253 unique human genes. Of the 253 human genes homologs, 209 genes showed expression in the PSOR data set. The genes were then filtered for average expression level across each cell type (as determined by Seurat clustering) in PSOR data set. Clusters were labeled as the following cell types: Keratinocytes (KCs, containing *KRT10*, *KRT15*, *KRT2*), Melanocytes (Mel, containing *PMEL*), T cells (TC, containing *CD3D*+), myeloid lineage (containing *LYZ*+), and an unknown cluster KRT23-MMP7 (*KRT23*+, *KRT18*+, *MMP7*+); 11 genes were found to have higher expression in keratinocytes (KC) than in myeloid-lineage population. The “TAR11” composite score of cells, representing the average expression level of these 12 genes in each cell, was then calculated. A comparative analysis was conducted between the expression level of TAR11 and level of *KRT6A* and *KRT17* in each cell.

### Image quantification and statistical analyses

Quantitation of immunofluorescence images, PLA images, and western blots were performed using ImageJ and CellProfiler. In vivo neutrophil infiltration levels were calculated as the area fraction of Ly6g-positive staining in the TPA-treated skin (minimum threshold = 5,000) subtracted by background (area fraction of Ly6g-positive staining in acetone-treated skin at the same time point). Quantification of PKCα membrane localization was based on the intensity of the PKCα signal on a line profile (line width 20 μm) across adjacent cells. Fluorescence intensities were calculated as the signal intensity of regions of interest (ROIs) for several fields within tissue sections, and several tissue sections, across biological replicates (e.g., mice). Quantification of PLA dots in cells transfected with EGFP-tagged keratins was done using ImageJ. ROIs were drawn around individual cells, guided by the EGFP cytoplasmic signal. After setting the minimum threshold to 3,000 to minimize background noise, the number of PLA dots per EGFP-positive cell was calculated using ImageJ’s Analyze Particles function to automatically count particles larger than 0.3 μm^2^. For PLA images of untransfected cells, which lack cytoplasmic staining, CellProfiler was used for cell segmentation. Briefly, nuclei were identified as primary objects using the DAPI signal. Cell boundaries were estimated as secondary objects, taking several parameters into account: nuclei propagation, an expansion up to 10 μm from the nuclear periphery using the Distance-B method, and differentiation between intracellular noise and the background signal in the extracellular space. The software then automatically counted the number of PLA particles larger than 0.3 μm^2^ per cell. For western blot images, normalized protein levels were calculated by dividing the integrated density of target protein band by the density of the housekeeping protein band. To quantify TUNEL-positive cells, the number of positively stained cells was counted as well as the total number of cells based on DAPI staining. The average percentage of positive cells was then calculated across multiple ROIs for each biological replicate. Quantification of mean intensity of desmoplakin and E-cadherin was performed using CellProfiler. The cell–cell junctions were determined using E-cadherin staining, and the ROI (“cell boundary”) was set to the area of the cell junctions that expanded outward by 0.5 μm and contracted inward by 0.5 μm. Error bars on histograms represent standard error of the mean (SEM) across biological replicates. Statistical significance was determined by one-way ANOVA followed by Tukey’s multiple comparisons test, two-way ANOVA followed by Sidak’s multiple comparisons test, and paired or unpaired *t* test. Differences with *p* < 0.05 were considered statistically significant. All statistical analyses were performed using GraphPad Prism.

## Supporting information

S1 Fig TAR is not related to the skin barrier status and is driven by local signal(s) in situ. **(A)** Tissue sections of WT mice ear skin at baseline (no Tx) and 12 h after a single topical application of acetone, immunostained for Ly6g and K14 (nuclei stained with DAPI). Scale bars: 50 μm. epi, epidermis; derm, dermis; hf, hair follicle. Dashed lines depict the dermo-epidermal interface. **(B)** Trans-epidermal water loss measurements (TEWL) of mouse ears treated with a single dose of acetone or TPA. *n* = 6. Two-way ANOVA. **(C)** Strategy to test whether TAR is mediated by local or systemic signals. Dual TPA treatment was applied to either the same ear (“local”) or contra-lateral ears (“distant”), 24 h apart. Clipart is open source https://openclipart.org/detail/17622/simple-cartoon-mouse-1; Creative Commons CC0 1.0 Universal License (https://creativecommons.org/publicdomain/zero/1.0/). **(D)** Tissue sections from (C) were immunostained for Ly6g, K14, and nuclei (DAPI). Scale bars: 50 μm. **(E)** Quantitation of neutrophil fluorescence signal (surface area measurements) of data in C, *n* = 3 mice. Data reported as mean ± SEM. One-way ANOVA. The source data used to derive the numerical values reported here can be found in **S1 Data**.
(PDF)

S2 Fig Additional analyses of the K17-dependent skin response to TPA treatments in vivo. **(A)** Sections from WT and *Krt17-/-* mouse skin treated with acetone or single TPA were immunostained for Ly6g, K14, and nuclei (DAPI). Scale bars: 50 μm. epi, epidermis; derm, dermis; hf, hair follicle. Dashed lines depict the dermo-epidermal interface. **(B)** TUNEL staining of WT and *Krt17-/-* mouse ear skin 6 h after either double TPA Tx (24 h apart) or TPA-UVB combination Tx (24 h apart). Scale bar: 50 μm**. (C)** Percentage of TUNEL positive cells in epidermis after double TPA (data from B). *n* = 3 mice. Data are shown as mean ± SEM. Unpaired *t* test. **(D, E)** Mouse ear tissues were harvested 6 h after double acetone or dual TPA Tx, 24 h apart, and processed for bulk RNAseq analysis. Volcano plots reporting on changes in mRNA transcripts levels in TPA-Tx vs. vehicle-Tx for (D) WT skin and (E) *Krt17-/-* skin. Genes that are significantly up- or down-regulated by 8-fold or more (adj. *P* < 0.01) are highlighted in blue (WT) or red (*Krt17-/-*). **(F)** Comparison of genes significantly down-regulated after dual TPA-Tx vs. dual acetone-Tx in WT and *Krt17-/-* mouse skin (Cutoffs: FDR-adjusted *P* < 0.01, fold change > 8). **(G)** Panther overrepresentation test using Reactome pathways (FDR-adjusted *P* < 0.05) for the 268 genes showing significant up-regulated expression in both WT and *Krt17-/-* skin after double TPA Tx (see Fig 2I). The source data used to derive the numerical values reported here can be found in **S1 Data**.
(PDF)

S3 Fig Ex vivo analyses of K17-dependent neutrophil recruitment. **(A)** Parental and *KRT17* null A431 keratinocytes stained for K17 and nuclei (DAPI). Scale bars: 20 μm. **(B)** Migration of human primary neutrophils towards A431 conditioned medium (CM) “titrated” (i.e., diluted) as indicated using control medium (*n* = 2). **(C)** Migration of human primary neutrophils towards CM from GFP-transfected parental A431, GFP-transfected *KRT17* null A431, and GFP-K17-transfected *KRT17* null A431 cells. Individual symbols depict data using neutrophils from different donors (*n* = 6). Data reported as mean ± SEM. One-way ANOVA. **(D)** Transcript levels of CXCR3 in human primary neutrophils before (t = 0) and after treatment with buffer (mHBSS, 2 h), culture medium control (2 h), CM from acetone-Tx parental A431 (2 h), or CM from TPA-Tx parental A431 (2 h). *n* = 3. Data reported as mean ± SEM. One-way ANOVA. **(E, F)** ELISA measurements for selected chemokine and cytokine levels in A431 CM (reported as pg/ml). *n* = 3 for CXCL2 (2 technical replicates each), and *n* = 4 for CXCL9 (4 technical replicates for 2 measurements, 2 technical replicates for the other 2 measurements). Data reported as mean ± SEM. One-way ANOVA. **(G)** ELISA measurements of TNFα levels in A431 CM (pg/ml). *n* = 2 measurements (4 technical replicates each). Data are shown as mean ± SEM. One-way ANOVA. Acet, Acetone. **(H)** Migration of human primary neutrophils towards DMSO (vehicle control) or fMLF, with or without the addition of a CXCR2 antagonist, a CXCR3 antagonist, and infliximab (anti-TNFα). *n* = 5. Data reported as mean ± SEM. Paired *t* test. The source data used to derive the numerical values reported here can be found in **S1 Data**.
(PDF)

S4 Fig TAR triggered by TPA-UVB dual treatment in mouse skin is partially PKCα-dependent. **(A)** Quantitation of total PKCα levels in epidermis of WT and *Krt17-/-* mouse skin 6 h after dual TPA Tx, 24 h apart. *n* = 3 mice. Data reported as mean ± SEM. One-way ANOVA. **(B)** WT and *Krt17-/-* mouse ears were treated with TPA followed by UVB 24 h later. Tissues were collected 6 h after UVB and immunostained for phosphor-PKCα (T638), K14, and nuclei (DAPI). Scale bars: 50 μm. epi, epidermis; derm, dermis; hf, hair follicle. Dashed lines depict the dermo-epidermal interface. **(C)** Quantitation of the phospho-PKCα signal in data from (B). *n* = 4 mice. Data are shown as mean ± SEM. One-way ANOVA. **(D)** Impact of pretreatment with the PKCα inhibitor Go6976 30 min prior to the 2nd Tx, UVB (24 h interval), in WT mouse ear skin. Sections were immunostained for Ly6g, K17, K14, and nuclei (DAPI). Scale bars: 50 μm. Asterisks denote the loss of epidermis-proximal pool of neutrophils when pretreated with Go6976. **(E, F)** Quantitation of neutrophil fluorescence signals using surface area measurements (E) and line vectors extending from the basement membrane to the bottom of the dermis (F). *n* = 3 mice. Data reported as mean ± SEM. One-way ANOVA. The source data used to derive the numerical values reported here can be found in **S1 Data**.
(PDF)

S5 Fig Effect of TPA treatment on desmosome and protein solubility in A431 keratinocytes. **(A)** Parental and *KRT17* null A431 cells were stained for desmoplakin, E-cadherin, and nuclei (DAPI) 30 min after acetone-control Tx, or 30 and 60 min after TPA Tx. Scale bars: 10 μm. Arrows point to desmoplakin staining at cell–cell borders, and arrows with asterisks denote partial loss of desmoplakin staining at cell–cell borders after TPA Tx. **(B, C)** Quantification of desmoplakin and E-cadherin staining at cell boundary (ROI width = 1 μm, see Methods). Each dot represents a cell. Data are shown as mean ± SEM. Two-way ANOVA. **(D)** Western blot analysis of the solubility of K17, PKCα, RACK1, and (control) β-Actin in parental A431 cells 1 h after TPA Tx, and 20 μg of total protein was loaded in each lane for the low-salt and triton-soluble fractions, and 2 μg of total protein was loaded in each lane for the triton-insoluble fractions. The source data used to derive the numerical values reported here can be found in **S1 Data**.
(PDF)

S6 Fig A TAR transcriptional signature occurs in stressed keratinocytes from human inflammatory skin diseases (complement to Fig 8). **(A)** Strategy for (i) devising a TAR-related signature from analyses of RNAseq data in dual TPATx mouse skin and (ii) harmonizing it with the human transcriptome using an existing data set from individuals with psoriasis (PSOR) [55] (see Methods). The set of 441 genes significantly up-regulated (>8-fold; adj *P* < 0.01) in WT, but not in *Krt17-/-*, mouse skin 6 h after dual TPA Tx (see Fig 2I) were designated as K17-dependent TAR genes; 209 homologs were identified in the PSOR data set [55], forming the basis for a “TAR209” score, and 11 of these 209 genes are expressed at significant levels in PSOR keratinocytes, as opposed to other cell types (see B and C), forming the basis for the TAR11 composite score. **(B)** Expression of the “TAR209” score in specific cell clusters within the PSOR data set [55]. **(C)** Expression of the “TAR11” score, which is based on the 11 genes showing higher expression levels in keratinocytes within the PSOR data set (*ANGPTL4*, *IFITM1*, *IFI16*, *PHLDA2*, *KLK1*, *PDPN*, *AKR1B10*, *ACAT2*, *TUBB6*, *OAS1*, *HRH2*), in different cell clusters in this data set. **(D)** Distribution of TAR11 scores in all individual cells from the AD single cell data set [64]. **(E, F)** Violin plots reporting on the expression levels of *KRT6A* (E) and *KRT17* (F) in cells showing high, medium, and low TAR11 scores. Cut-offs were arbitrarily set at the 95th percentile for a high TAR score and at the 75th percentile for a medium TAR score based on the distributions shown in (D). **(G, I)** Sections of WT and *Krt17-/-* mouse ear skin collected 6 h after double TPA Tx (24 h apart) were immunostained for Histamine receptor H2 (HRH2) (G) or 2’-5’-Oligoadenylate Synthetase 1 (OAS1) (I). Scale bars: 50 μm. Dashed lines depict the dermo-epidermal interface. **(H, J)** Quantitation of the signal for HRH2 (H) or OAS1 (J) levels in epidermis from G or I. *n* = 3 mice. Data are shown as mean ± SEM. One-way ANOVA. The source data used to derive the numerical values reported here can be found in **S1 Data**.
(PDF)

S1 Table List of antibodies used in this study. Antibodies utilized in immunofluorescence, proximity ligation assay (PLA), and western blotting.
(XLSX)

S2 Table Oligonucleotide primer sequences used when making K17-K14 chimeric cDNAs and conducting qRT-PCR assays. Legend: K14h-K17r-K17t: Chimeric construct consisting of the head domain of K14 and the rod and tail domains of K17; K17h-K14r-K14t: Chimeric construct consisting of the head domain of K17 and the rod and tail domains of K14.
(XLSX)

S3 Table ELISA data for chemokines and cytokines analyzed in conditioned medium (CM) from A431 keratinocytes. Corresponding sample numbers indicating CM collected from the same experimental batch. Legend: *Blue: Values below limit of detection; *Red: Values above the highest standards.
(XLSX)

S4 Table Genes significantly up-regulated at 6 h after dual TPA TX in WT and *Krt17-/-* mouse skin. Complete list of significantly up-regulated genes at 6 h after dual TPA treatment of ear skin, 24 h apart, in WT and *Krt17-/-* mice. (Cutoff: Adjusted *P* < 0.01, Fold change>8).
(XLSX)

S5 Table Genes significantly down-regulated at 6 h after dual TPA TX in WT and *Krt17-/-* mouse skin. Complete list of significantly down-regulated genes at 6 h after dual TPA treatment of ear skin, 24 h apart, in WT and *Krt17-/-* mice. (Cutoff: Adjusted *P* < 0.01, Fold change>8).
(XLSX)

S6 Table Identification of 253 human gene homologs corresponding to the K17-dependent TAR genes identified in mice. The 441 genes that are up-regulated >8-fold (adj. *P* < 0.01) in the bulk RNAseq data set in WT, but not in *Krt17-/-*, mouse skin at 6 h after dual TPA Tx (24 h apart) were set aside as K17-dependent TAR genes. Their corresponding human gene homologs (253 genes) were identified and are listed in this table.
(XLSX)

S7 Table Identification of the TAR11 transcriptomic signature. A total of 209 genes from the 253 TAR-related human homologs (see **S6 Table**) were found to occur in the PSOR single-cell RNAseq data set (see Cheng and colleagues, [55]), and 11 of these genes show preferential expression in keratinocytes (KC) over the myeloid-lineage cells (see **S6C Fig**). These 11 genes form the basis for the “TAR11” composite score.
(XLSX)

S8 Table Changes in TAR11 gene expression in mouse ear skin subjected to single vs. double TPA Tx in WT mice bulk RNA seq data sets. Fold changes for the mouse homologs corresponding to the human TAR11 gene signature following double TPA Tx (this study) or single TPA Tx (from Abdallah and colleagues, [65]) in mouse ear skin (source data: bulk RNAseq data sets from each study); 7 of these genes, highlighted in bold, showed exclusive or notably greater up-regulation with double TPA relative to single TPA treatment. The remaining 4 genes in the TRA11 group were either up-regulated to a similar degree in mouse ear skin subjected to single vs. double TPA Tx or were not detected in RNA-seq data set after a single TPA Tx.
(XLSX)

S1 Raw Images Raw images. (PDF)

S1 Data Source data. (XLSX)

## Abbreviations

AD: atopic dermatitis
ATCC: American Type Culture Collection
BSA: bovine serum albumin
cDNA: complementary DNA
CM: conditioned medium
HS: hidradenitis suppurativa
IF: intermediate filament
IP: immunoprecipitation
K17: Keratin 17
PC: pachyonychia congenita
PFA: paraformaldehyde
PKCα: protein kinase C alpha
PLA: proximity ligation assay
PM: plasma membrane
PSOR: psoriasis
PTM: posttranslational modification
RACK1: Receptor for Activated C Kinase 1
ROI: region of interest
SNN: shared nearest neighbor
TAR: transient amplification response
TEWL: trans-epidermal water loss
Tx: treatment
