# Supplementary material for: Repeated stress to the skin amplifies neutrophil infiltration in a keratin 17- and PKCα-dependent manner
Source: PLoS Biol. 2024 Aug 19;22(8):e3002779. doi: 10.1371/journal.pbio.3002779 (PMC11361748; doi:10.1371/journal.pbio.3002779)
Supplement: S1 Fig — (A) Tissue sections of WT mice ear skin at baseline (no Tx) and 12 h after a single topical application of acetone, immunostained for Ly6g and K14 (nuclei stained with DAPI). Scale bars: 50 μm. epi, epidermis; derm, dermis; hf, hair follicle. Dashed lines depict the dermo-epidermal interface. (B) Trans-epidermal water loss measurements (TEWL) of mouse ears treated with a single dose of acetone or TPA. n = 6. Two-way ANOVA. (C) Strategy to test whether TAR is mediated by local or systemic signals. Dual TPA treatment was applied to either the same ear (“local”) or contra-lateral ears (“distant”), 24 h apart. Clipart is open source https://openclipart.org/detail/17622/simple-cartoon-mouse-1; Creative Commons CC0 1.0 Universal License (https://creativecommons.org/publicdomain/zero/1.0/). (D) Tissue sections from (C) were immunostained for Ly6g, K14, and nuclei (DAPI). Scale bars: 50 μm. (E) Quantitation of neutrophil fluorescence signal (surface area measurements) of data in C, n = 3 mice. Data reported as mean ± SEM. One-way ANOVA. The source data used to derive the numerical values reported here can be found in S1 Data. (PDF) [file pbio.3002779.s001.pdf]

Supplemental Figure 1. Xu et al.

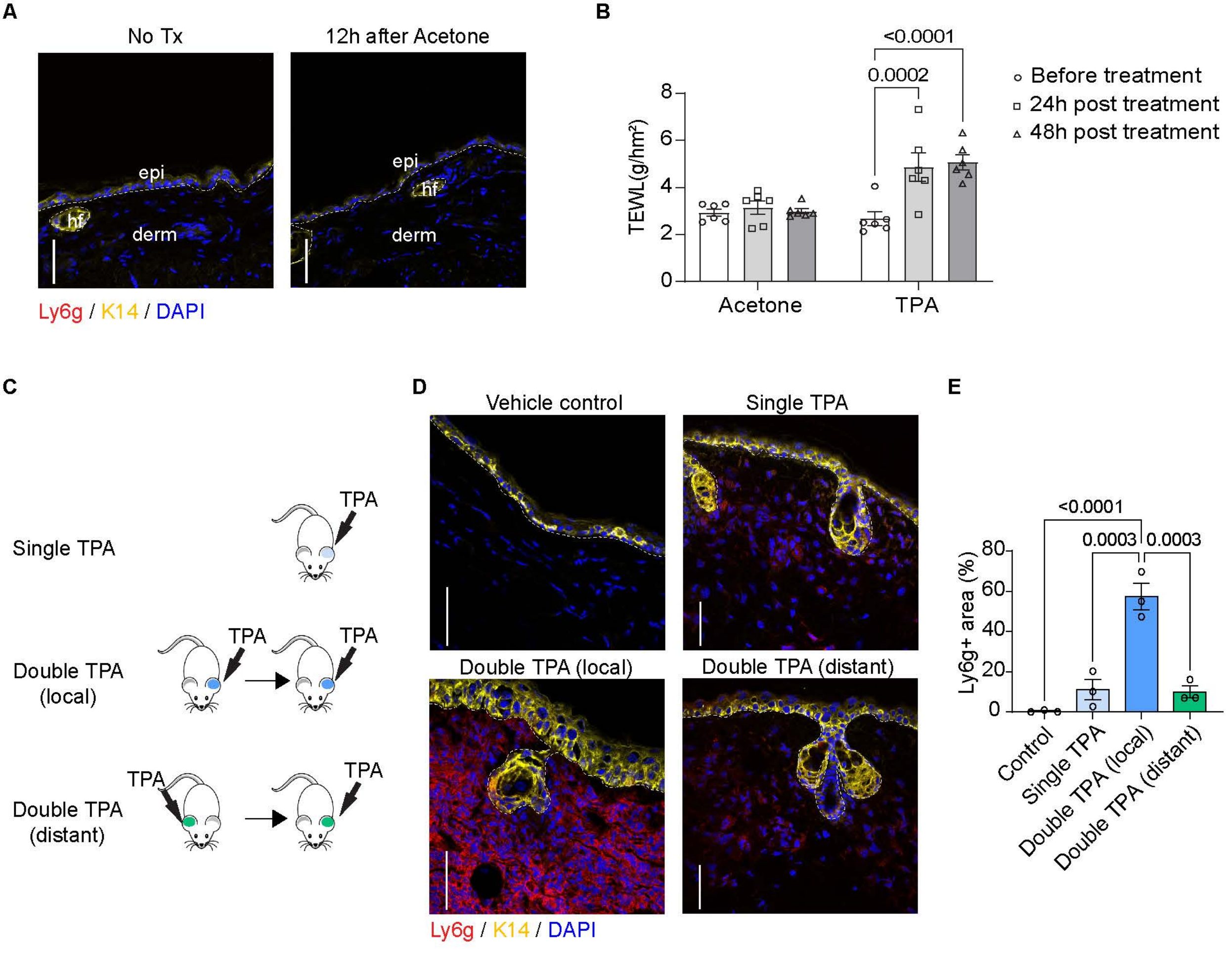

Supplemental Figure 1 (Xu *et al.*).

TAR is not related to the skin barrier status and is driven by local signal(s) *in situ.*

A) Tissue sections of WT mice ear skin at baseline (no Tx) and 12h after a single topical application of acetone, immunostained for Ly6g and K14 (nuclei stained with DAPI). Scale bars: 50μm. epi, epidermis; derm, dermis; hf, hair follicle. Dashed lines depict the dermo-epidermal interface. **B)** Trans-epidermal water loss measurements (TEWL) of mouse ears treated with a single dose of acetone or TPA. n=6. Two-way ANOVA. **C)** Strategy to test whether TAR is mediated by local or systemic signals. Dual TPA treatment was applied to either the same ear ("local") or contra-lateral ears ("distant"), 24h apart. Clipart is open source https://openclipart.org/detail/17622/simple-cartoon-mouse-1; Creative Commons CC0 1.0 Universal License (https://creativecommons.org/publicdomain/zero/1.0/). **D)** Tissue sections from C) were immunostained for Ly6g, K14, and nuclei (DAPI). Scale bars: 50μm. **E)** Quantitation of neutrophil fluorescence signal (surface area measurements) of data in C, n=3 mice. Data reported as mean ± SEM. One-way ANOVA. The source data used to derive the numerical values reported here can be found in "Data S1".
