# Supplementary material for: Repeated stress to the skin amplifies neutrophil infiltration in a keratin 17- and PKCα-dependent manner
Source: PLoS Biol. 2024 Aug 19;22(8):e3002779. doi: 10.1371/journal.pbio.3002779 (PMC11361748; doi:10.1371/journal.pbio.3002779)
Supplement: S2 Fig — (A) Sections from WT and Krt17-/- mouse skin treated with acetone or single TPA were immunostained for Ly6g, K14, and nuclei (DAPI). Scale bars: 50 μm. epi, epidermis; derm, dermis; hf, hair follicle. Dashed lines depict the dermo-epidermal interface. (B) TUNEL staining of WT and Krt17-/- mouse ear skin 6 h after either double TPA Tx (24 h apart) or TPA-UVB combination Tx (24 h apart). Scale bar: 50 μm. (C) Percentage of TUNEL positive cells in epidermis after double TPA (data from B). n = 3 mice. Data are shown as mean ± SEM. Unpaired t test. (D, E) Mouse ear tissues were harvested 6 h after double acetone or dual TPA Tx, 24 h apart, and processed for bulk RNAseq analysis. Volcano plots reporting on changes in mRNA transcripts levels in TPA-Tx vs. vehicle-Tx for (D) WT skin and (E) Krt17-/- skin. Genes that are significantly up- or down-regulated by 8-fold or more (adj. P < 0.01) are highlighted in blue (WT) or red (Krt17-/-). (F) Comparison of genes significantly down-regulated after dual TPA-Tx vs. dual acetone-Tx in WT and Krt17-/- mouse skin (Cutoffs: FDR-adjusted P < 0.01, fold change > 8). (G) Panther overrepresentation test using Reactome pathways (FDR-adjusted P < 0.05) for the 268 genes showing significant up-regulated expression in both WT and Krt17-/- skin after double TPA Tx (see Fig 2I). The source data used to derive the numerical values reported here can be found in S1 Data. (PDF) [file pbio.3002779.s002.pdf]

Supplemental Figure 2. Xu et al.

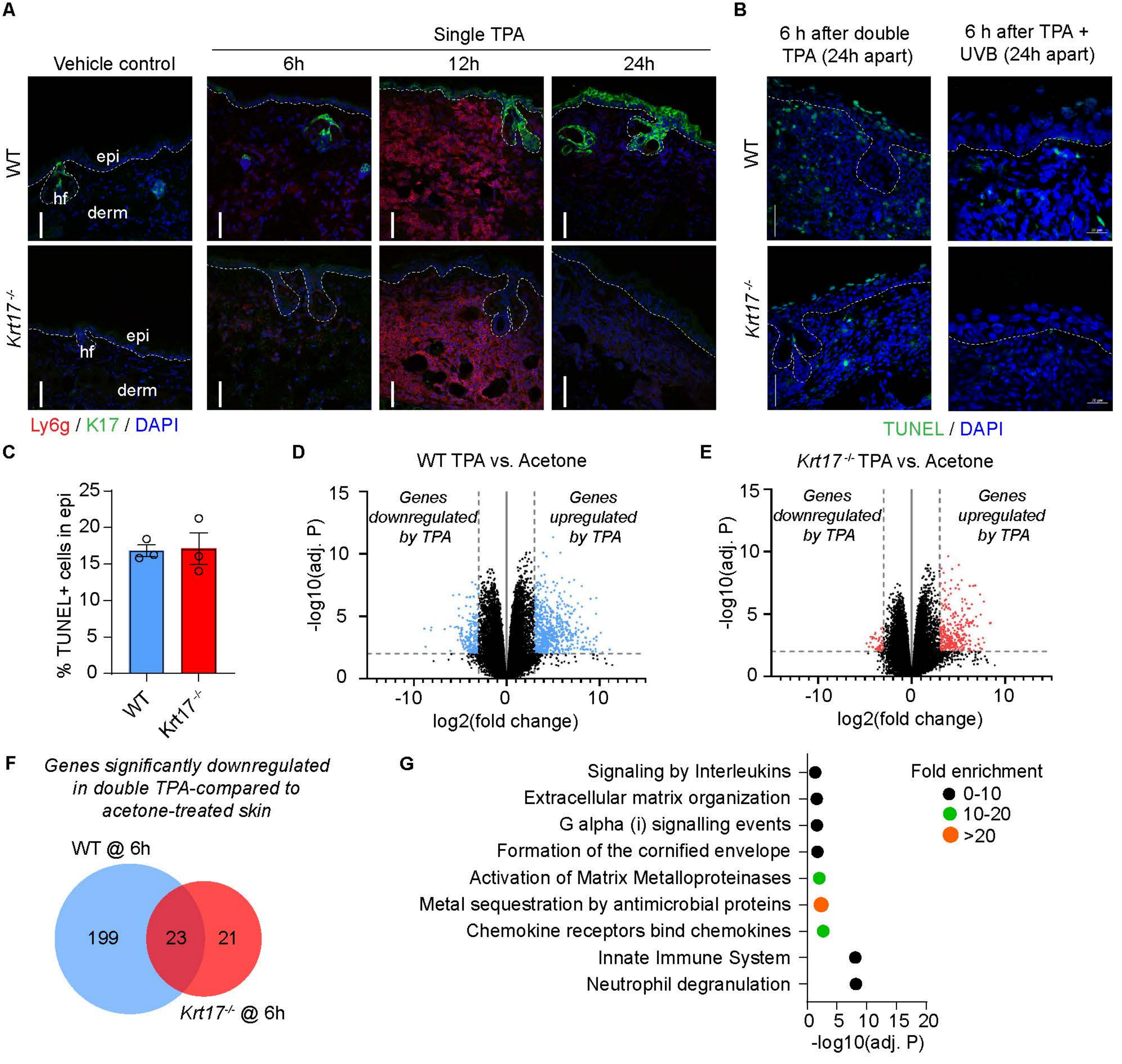

__Supplemental Figure 2__ **(Xu *et al.*).**

Additional analyses of the K17-dependent skin response to TPA treatments *in vivo.*

A) Sections from WT and *Krt17*$^{-/-}$ mouse skin treated with acetone or single TPA were immuno-stained for Ly6g, K14, and nuclei (DAPI). Scale bars: 50µm. epi, epidermis; derm, dermis; hf, hair follicle. Dashed lines depict the dermo-epidermal interface. **B)** TUNEL staining of WT and *Krt17*$^{-/-}$ mouse ear skin 6h after either double TPA Tx (24h apart) or TPA-UVB combination Tx (24h apart). Scale bar: 50µm**. C)** Percentage of TUNEL positive cells in epidermis after double TPA (data from B). n=3 mice. Data are shown as mean ± SEM. Unpaired t-test. **D-E)** Mouse ear tissues were harvested 6h after double acetone or dual TPA Tx, 24h apart, and processed for bulk RNAseq analysis. Volcano plots reporting on changes in mRNA transcripts levels in TPA-Tx vs. vehicle-Tx for D) WT skin and E) *Krt17*$^{-/-}$ skin. Genes that are significantly up- or down-regulated by 8-fold or more (adj. P < 0.01) are highlighted in blue (WT) or red (*Krt17*$^{-/-}$).

F) Comparison of genes significantly downregulated after dual TPA-Tx vs. dual acetone-Tx in WT and *Krt17*$^{-/-}$ mouse skin (Cutoffs: FDR-adjusted P < 0.01, fold change > 8). **G)** Panther over-representation test using Reactome pathways (FDR-adjusted P < 0.05) for the 268 genes showing significant upregulated expression in both WT and *Krt17*$^{-/-}$ skin after double TPA Tx (see Figure 2I). The source data used to derive the numerical values reported here can be found in “Data S1”.
