# Supplementary material for: Repeated stress to the skin amplifies neutrophil infiltration in a keratin 17- and PKCα-dependent manner
Source: PLoS Biol. 2024 Aug 19;22(8):e3002779. doi: 10.1371/journal.pbio.3002779 (PMC11361748; doi:10.1371/journal.pbio.3002779)
Supplement: S3 Fig — (A) Parental and KRT17 null A431 keratinocytes stained for K17 and nuclei (DAPI). Scale bars: 20 μm. (B) Migration of human primary neutrophils towards A431 conditioned medium (CM) “titrated” (i.e., diluted) as indicated using control medium (n = 2). (C) Migration of human primary neutrophils towards CM from GFP-transfected parental A431, GFP-transfected KRT17 null A431, and GFP-K17-transfected KRT17 null A431 cells. Individual symbols depict data using neutrophils from different donors (n = 6). Data reported as mean ± SEM. One-way ANOVA. (D) Transcript levels of CXCR3 in human primary neutrophils before (t = 0) and after treatment with buffer (mHBSS, 2 h), culture medium control (2 h), CM from acetone-Tx parental A431 (2 h), or CM from TPA-Tx parental A431 (2 h). n = 3. Data reported as mean ± SEM. One-way ANOVA. (E, F) ELISA measurements for selected chemokine and cytokine levels in A431 CM (reported as pg/ml). n = 3 for CXCL2 (2 technical replicates each), and n = 4 for CXCL9 (4 technical replicates for 2 measurements, 2 technical replicates for the other 2 measurements). Data reported as mean ± SEM. One-way ANOVA. (G) ELISA measurements of TNFα levels in A431 CM (pg/ml). n = 2 measurements (4 technical replicates each). Data are shown as mean ± SEM. One-way ANOVA. Acet, Acetone. (H) Migration of human primary neutrophils towards DMSO (vehicle control) or fMLF, with or without the addition of a CXCR2 antagonist, a CXCR3 antagonist, and infliximab (anti-TNFα). n = 5. Data reported as mean ± SEM. Paired t test. The source data used to derive the numerical values reported here can be found in S1 Data. (PDF) [file pbio.3002779.s003.pdf]

Supplemental Figure 3. Xu et al.

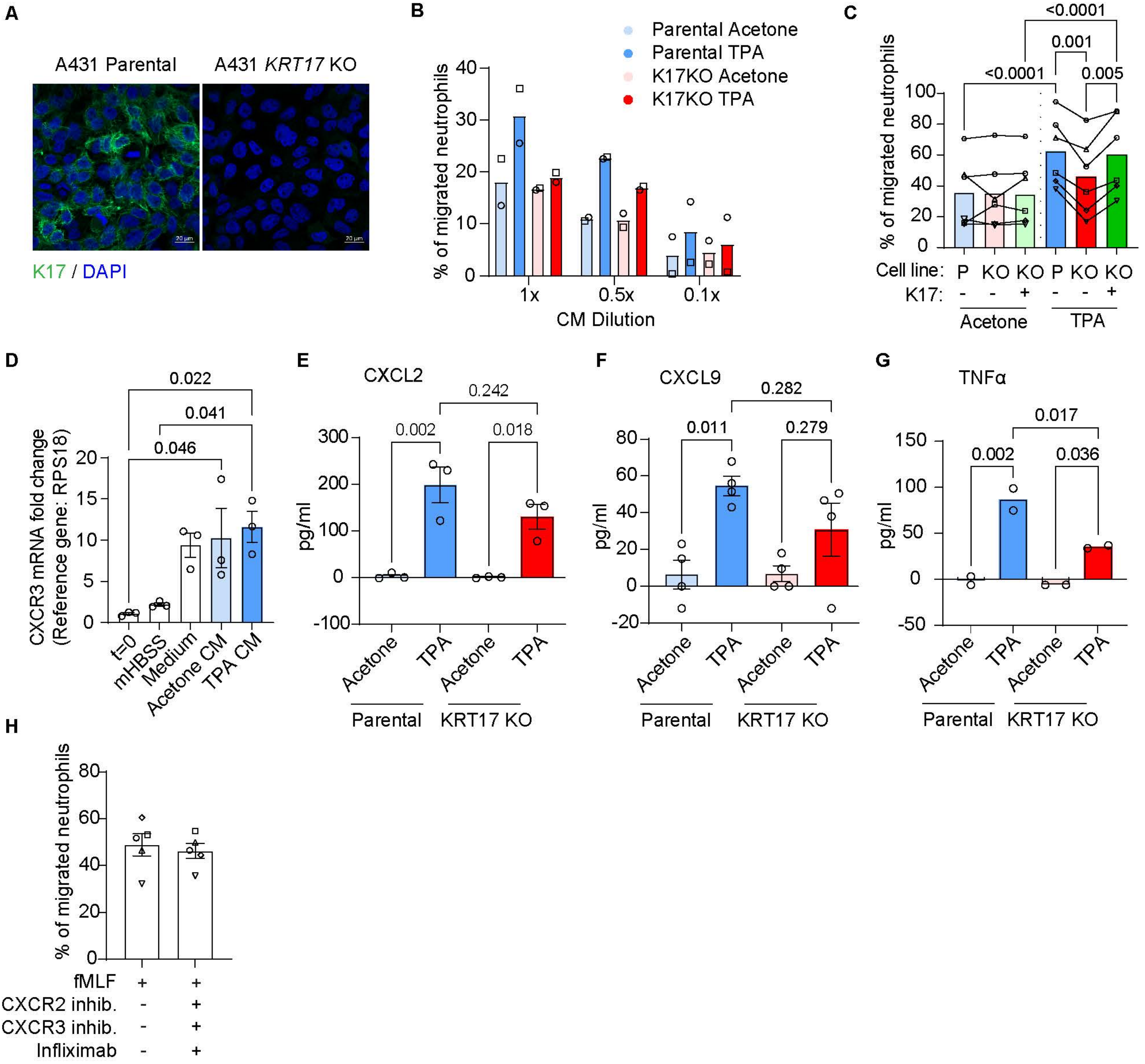

**<u>Supplemental Figure 3</u> (Xu *et al.*).**

***Ex vivo* analyses of K17-dependent neutrophil recruitment.**

**A)** Parental and *KRT17* null A431 keratinocytes stained for K17 and nuclei (DAPI). Scale bars: 20μm. **B)** Migration of human primary neutrophils towards A431 conditioned medium (CM) "titrated" (i.e., diluted) as indicated using control medium (n=2). **C)** Migration of human primary neutrophils towards CM from GFP-transfected parental A431, GFP-transfected *KRT17* null A431, and GFP-K17-transfected *KRT17* null A431 cells. Individual symbols depict data using neutrophils from different donors (n=6). Data reported as mean ± SEM. One-way ANOVA. **D)** Transcript levels of CXCR3 in human primary neutrophils before (t=0) and after treatment with buffer (mHBSS, 2h), culture medium control (2h), CM from acetone-Tx parental A431 (2h), or CM from TPA-Tx parental A431 (2h). n=3. Data reported as mean ± SEM. One-way ANOVA. **E-F)** ELISA measurements for selected chemokine and cytokine levels in A431 CM (reported as pg/ml). n=3 for CXCL2 (2 technical replicates each), and n=4 for CXCL9 (4 technical replicates for 2 measurements, 2 technical replicates for the other 2 measurements). Data reported as mean ± SEM. One-way ANOVA. **G)** ELISA measurements of TNF$\alpha$ levels in A431 CM (pg/ml). n=2 measurements (4 technical replicates each). Data are shown as mean ± SEM. One-way ANOVA. Acet, Acetone. **H)** Migration of human primary neutrophils towards DMSO (vehicle control) or fMLF, with or without the addition of a CXCR2 antagonist, a CXCR3 antagonist, and infliximab (anti-TNF$\alpha$). n=5. Data reported as mean ± SEM. Paired t-test. The source data used to derive the numerical values reported here can be found in "Data S1".
