# Supplementary material for: Repeated stress to the skin amplifies neutrophil infiltration in a keratin 17- and PKCα-dependent manner
Source: PLoS Biol. 2024 Aug 19;22(8):e3002779. doi: 10.1371/journal.pbio.3002779 (PMC11361748; doi:10.1371/journal.pbio.3002779)
Supplement: S4 Fig — (A) Quantitation of total PKCα levels in epidermis of WT and Krt17-/- mouse skin 6 h after dual TPA Tx, 24 h apart. n = 3 mice. Data reported as mean ± SEM. One-way ANOVA. (B) WT and Krt17-/- mouse ears were treated with TPA followed by UVB 24 h later. Tissues were collected 6 h after UVB and immunostained for phosphor-PKCα (T638), K14, and nuclei (DAPI). Scale bars: 50 μm. epi, epidermis; derm, dermis; hf, hair follicle. Dashed lines depict the dermo-epidermal interface. (C) Quantitation of the phospho-PKCα signal in data from (B). n = 4 mice. Data are shown as mean ± SEM. One-way ANOVA. (D) Impact of pretreatment with the PKCα inhibitor Go6976 30 min prior to the 2nd Tx, UVB (24 h interval), in WT mouse ear skin. Sections were immunostained for Ly6g, K17, K14, and nuclei (DAPI). Scale bars: 50 μm. Asterisks denote the loss of epidermis-proximal pool of neutrophils when pretreated with Go6976. (E, F) Quantitation of neutrophil fluorescence signals using surface area measurements (E) and line vectors extending from the basement membrane to the bottom of the dermis (F). n = 3 mice. Data reported as mean ± SEM. One-way ANOVA. The source data used to derive the numerical values reported here can be found in S1 Data. (PDF) [file pbio.3002779.s004.pdf]

Supplemental Figure 4. Xu et al.

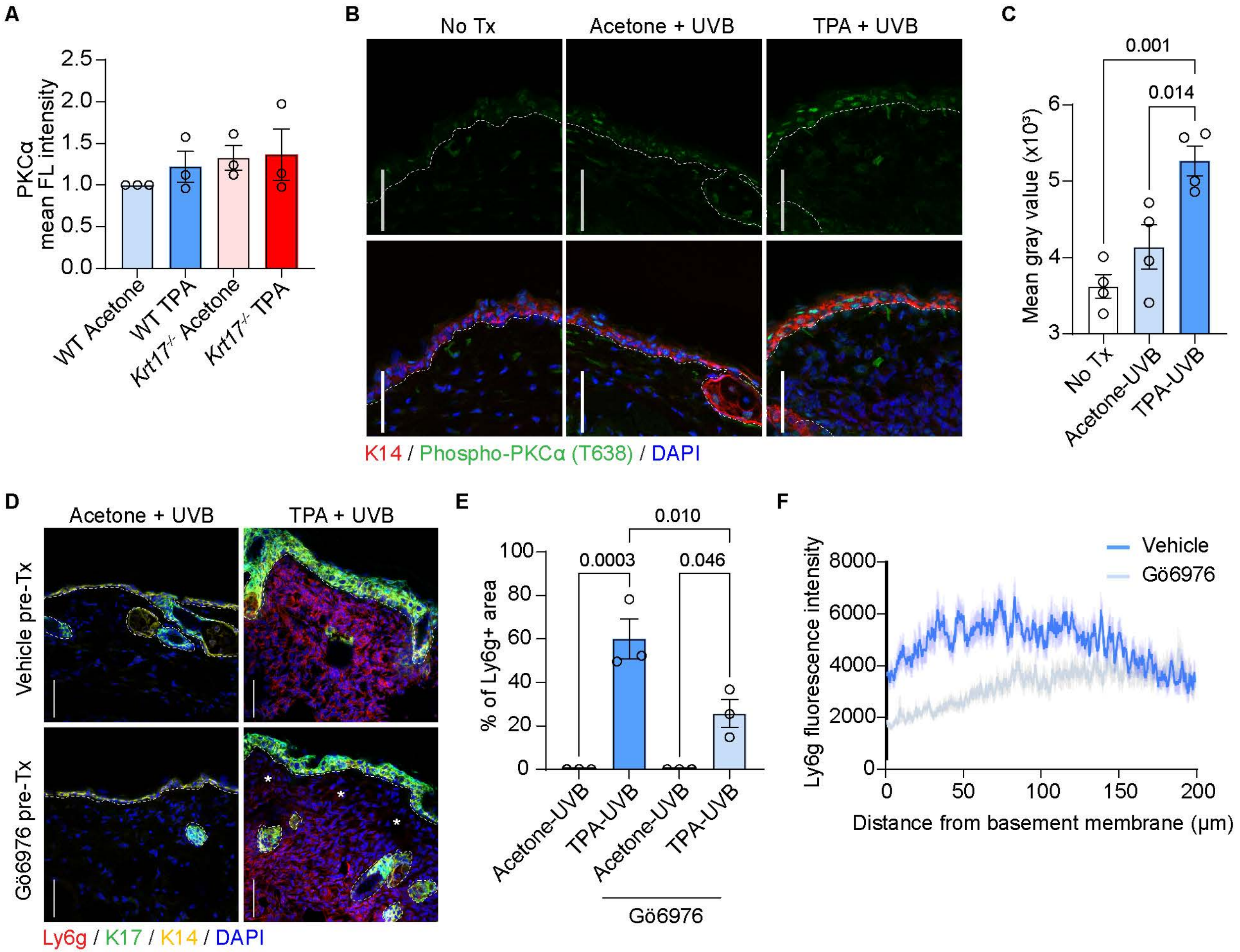

Supplemental Figure 4 (**Xu *et al.***).

TAR triggered by TPA-UVB dual treatment in mouse skin is partially PKCα-dependent.

A) Quantitation of total PKCα levels in epidermis of WT and *Krt17*$^{-/-}$ mouse skin 6h after dual TPA Tx, 24h apart. n=3 mice. Data reported as mean ± SEM. One-way ANOVA. **B)** WT and *Krt17*$^{-/-}$ mouse ears were treated with TPA followed by UVB 24h later. Tissues were collected 6h after UVB and immunostained for phosphor-PKCα (T638), K14, and nuclei (DAPI). Scale bars: 50μm. epi, epidermis; derm, dermis; hf, hair follicle. Dashed lines depict the dermo-epidermal interface. **C)** Quantitation of the phospho-PKCα signal in data from B. n=4 mice. Data are shown as mean ± SEM. One-way ANOVA. **D)** Impact of pre-treatment with the PKCα inhibitor Gö6976 30min prior to the 2nd Tx, UVB (24h interval), in WT mouse ear skin. Sections were immunostained for Ly6g, K17, K14, and nuclei (DAPI). Scale bars: 50μm. Asterisks denote the loss of epidermis-proximal pool of neutrophils when pretreated with Gö6976. **E-F)** Quantitation of neutrophil fluorescence signals using surface area measurements (E) and line vectors extending from the basement membrane to the bottom of the dermis (F). n=3 mice. Data reported as mean ± SEM. One-way ANOVA. The source data used to derive the numerical values reported here can be found in “Data S1”.
