# Supplementary material for: Repeated stress to the skin amplifies neutrophil infiltration in a keratin 17- and PKCα-dependent manner
Source: PLoS Biol. 2024 Aug 19;22(8):e3002779. doi: 10.1371/journal.pbio.3002779 (PMC11361748; doi:10.1371/journal.pbio.3002779)
Supplement: S5 Fig — (A) Parental and KRT17 null A431 cells were stained for desmoplakin, E-cadherin, and nuclei (DAPI) 30 min after acetone-control Tx, or 30 and 60 min after TPA Tx. Scale bars: 10 μm. Arrows point to desmoplakin staining at cell–cell borders, and arrows with asterisks denote partial loss of desmoplakin staining at cell–cell borders after TPA Tx. (B, C) Quantification of desmoplakin and E-cadherin staining at cell boundary (ROI width = 1 μm, see Methods). Each dot represents a cell. Data are shown as mean ± SEM. Two-way ANOVA. (D) Western blot analysis of the solubility of K17, PKCα, RACK1, and (control) β-Actin in parental A431 cells 1 h after TPA Tx, and 20 μg of total protein was loaded in each lane for the low-salt and triton-soluble fractions, and 2 μg of total protein was loaded in each lane for the triton-insoluble fractions. The source data used to derive the numerical values reported here can be found in S1 Data. (PDF) [file pbio.3002779.s005.pdf]

Xu et al. Supplemental figure 5

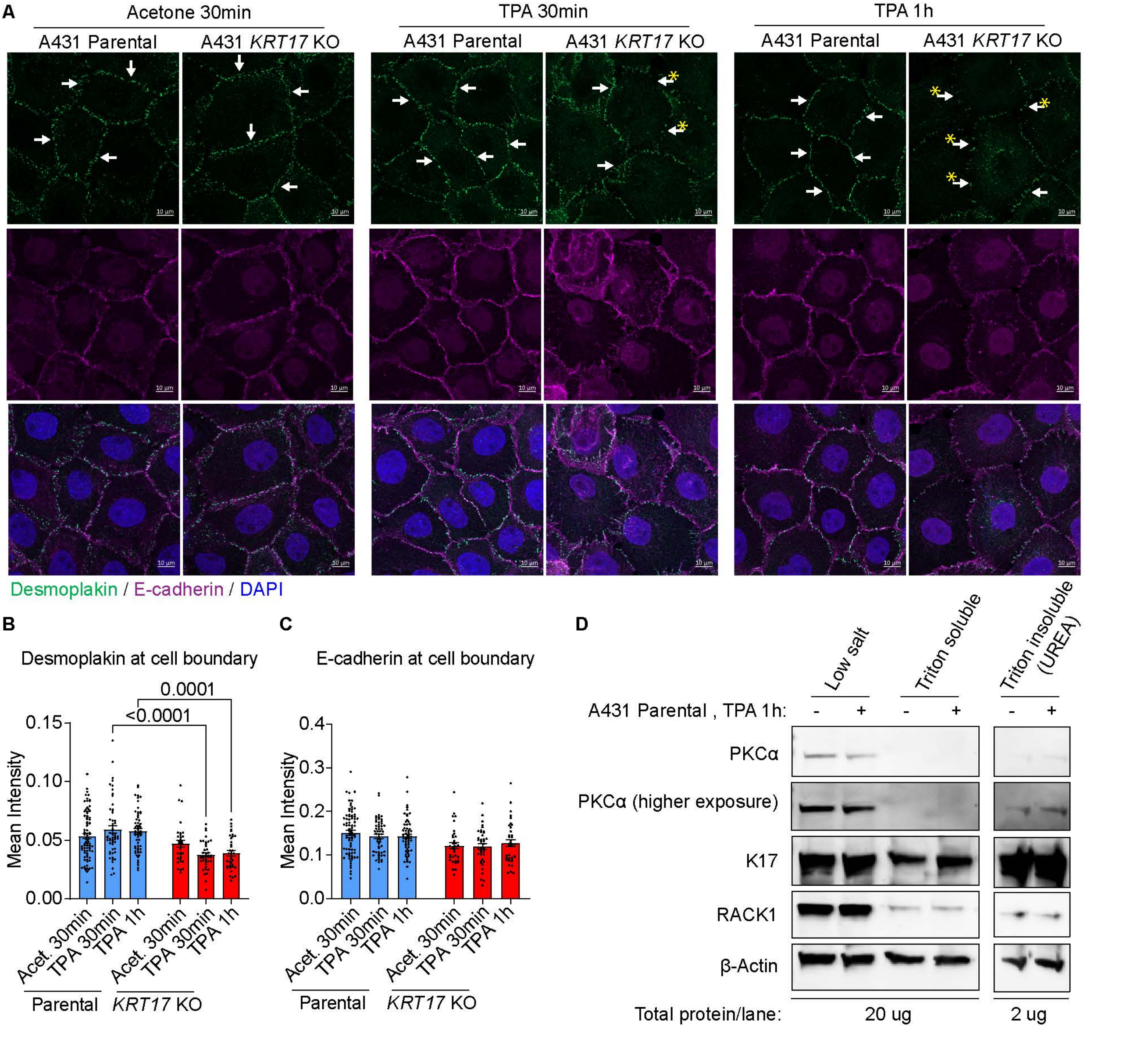

Supplemental Figure 5 (**Xu *et al.***).

Effect of TPA treatment on desmosome and protein solubility in A431 keratinocytes.

A) Parental and *KRT17* null A431 cells were stained for desmoplakin, E-cadherin, and nuclei (DAPI) 30 min after acetone-control Tx, or 30 and 60 min after TPA Tx. Scale bars: 10μm. Arrows point to desmoplakin staining at cell-cell borders, and arrows with asterisks denote partial loss of desmoplakin staining at cell-cell borders after TPA Tx. **B-C)** Quantification of desmoplakin and E-cadherin staining at cell boundary (ROI width=1μm, see Methods). Each dot represents a cell. Data are shown as mean ± SEM. Two-way ANOVA. **D)** Western blot analysis of the solubility of K17, PKCα, RACK1 and (control) β-Actin in parental A431 cells 1h after TPA Tx. 20μg of total protein was loaded in each lane for the low-salt and triton-soluble fractions, and 2μg of total protein was loaded in each lane for the triton-insoluble fractions. The source data used to derive the numerical values reported here can be found in “Data S1”.
