# Supplementary material for: Repeated stress to the skin amplifies neutrophil infiltration in a keratin 17- and PKCα-dependent manner
Source: PLoS Biol. 2024 Aug 19;22(8):e3002779. doi: 10.1371/journal.pbio.3002779 (PMC11361748; doi:10.1371/journal.pbio.3002779)
Supplement: S6 Fig — (A) Strategy for (i) devising a TAR-related signature from analyses of RNAseq data in dual TPATx mouse skin and (ii) harmonizing it with the human transcriptome using an existing data set from individuals with psoriasis (PSOR) [55] (see Methods). The set of 441 genes significantly up-regulated (>8-fold; adj P < 0.01) in WT, but not in Krt17-/-, mouse skin 6 h after dual TPA Tx (see Fig 2I) were designated as K17-dependent TAR genes; 209 homologs were identified in the PSOR data set [55], forming the basis for a “TAR209” score, and 11 of these 209 genes are expressed at significant levels in PSOR keratinocytes, as opposed to other cell types (see B and C), forming the basis for the TAR11 composite score. (B) Expression of the “TAR209” score in specific cell clusters within the PSOR data set [55]. (C) Expression of the “TAR11” score, which is based on the 11 genes showing higher expression levels in keratinocytes within the PSOR data set (ANGPTL4, IFITM1, IFI16, PHLDA2, KLK1, PDPN, AKR1B10, ACAT2, TUBB6, OAS1, HRH2), in different cell clusters in this data set. (D) Distribution of TAR11 scores in all individual cells from the AD single cell data set [64]. (E, F) Violin plots reporting on the expression levels of KRT6A (E) and KRT17 (F) in cells showing high, medium, and low TAR11 scores. Cut-offs were arbitrarily set at the 95th percentile for a high TAR score and at the 75th percentile for a medium TAR score based on the distributions shown in (D). (G, I) Sections of WT and Krt17-/- mouse ear skin collected 6 h after double TPA Tx (24 h apart) were immunostained for Histamine receptor H2 (HRH2) (G) or 2’-5’-Oligoadenylate Synthetase 1 (OAS1) (I). Scale bars: 50 μm. Dashed lines depict the dermo-epidermal interface. (H, J) Quantitation of the signal for HRH2 (H) or OAS1 (J) levels in epidermis from G or I. n = 3 mice. Data are shown as mean ± SEM. One-way ANOVA. The source data used to derive the numerical values reported here can be found in S1 Data. (PDF) [file pbio.3002779.s006.pdf]

Supplemental Figure 6. Xu et al.

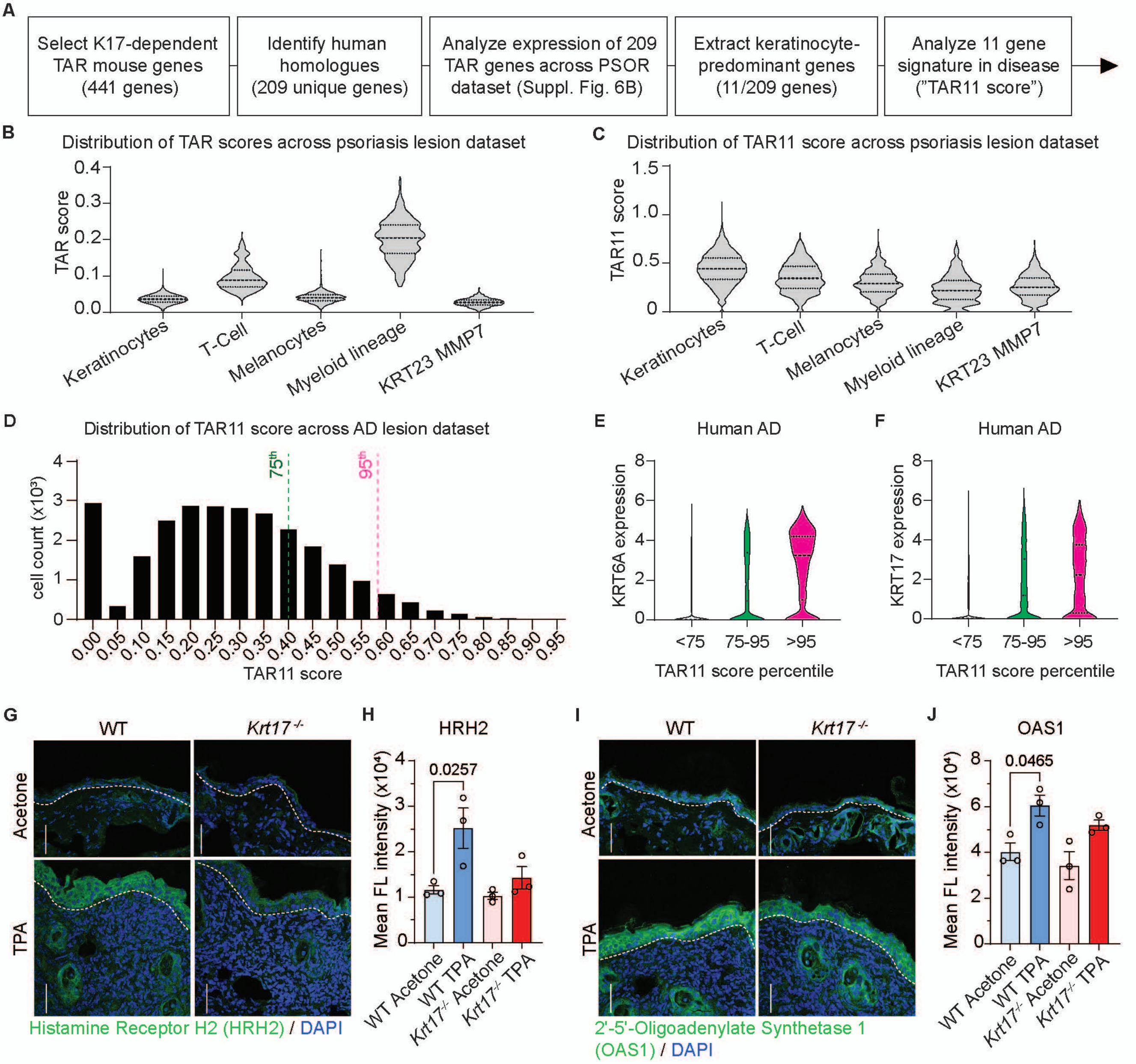

Supplemental Figure 6 (Xu *et al.*).

A TAR transcriptional signature occurs in stressed keratinocytes from human inflammatory skin diseases (complement to Figure 8).

A) Strategy for i) devising a TAR-related signature from analyses of RNAseq data in dual TPA-Tx mouse skin and ii) harmonizing it with the human transcriptome using an existing dataset from individuals with psoriasis (PSOR) (55) (see Methods). The set of 441 genes significantly upregulated (>8-fold; adj P<0.01) in WT, but not in *Krt17*$^{-/-}$, mouse skin 6h after dual TPA Tx (see Fig. 2I) were designated as K17-dependent TAR genes. 209 homologs were identified in the PSOR dataset (55), forming the basis for a “TAR209” score. 11 of these 209 genes are expressed at significant levels in PSOR keratinocytes, as opposed to other cell types (see B and C), forming the basis for the TAR11 composite score. **B)** Expression of the “TAR209” score in specific cell clusters within the PSOR dataset (55). **C)** Expression of the “TAR11” score, which is based on the 11 genes showing higher expression levels in keratinocytes within the PSOR dataset (*ANGPTL4, IFITM1, IFI16, PHLDA2, KLK1, PDPN, AKR1B10, ACAT2, TUBB6, OAS1, HRH2*), in different cell clusters in this dataset. **D)** Distribution of TAR11 scores in all individual cells from the AD single cell dataset (64). **E, F)** Violin plots reporting on the expression levels of *KRT6A* (E) and *KRT17* (F) in cells showing high, medium, and low TAR11 scores. Cut-offs were arbitrarily set at the 95th percentile for a high TAR score and at the 75th percentile for a medium TAR score based on the distributions shown in D. **G, I)** Sections of WT and *Krt17*$^{-/-}$ mouse ear skin collected 6h after double TPA Tx (24h apart) were immunostained for Histamine receptor H2 (HRH2) (G) or 2'-5'-Oligoadenylate Synthetase 1 (OAS1) (I). Scale bars: 50µm. Dashed lines depict the dermo-epidermal interface. **H, J)** Quantitation of the signal for HRH2 (H) or OAS1 (J) levels in epidermis from G or I. n=3 mice. Data are shown as mean ± SEM. One-way ANOVA. The source data used to derive the numerical values reported here can be found in “Data S1”.
