# Supplementary material for: Repeated stress to the skin amplifies neutrophil infiltration in a keratin 17- and PKCα-dependent manner
Source: PLoS Biol. 2024 Aug 19;22(8):e3002779. doi: 10.1371/journal.pbio.3002779 (PMC11361748; doi:10.1371/journal.pbio.3002779)
Supplement: S1 Raw Images — (PDF) [file pbio.3002779.s015.pdf]

S1_raw_images

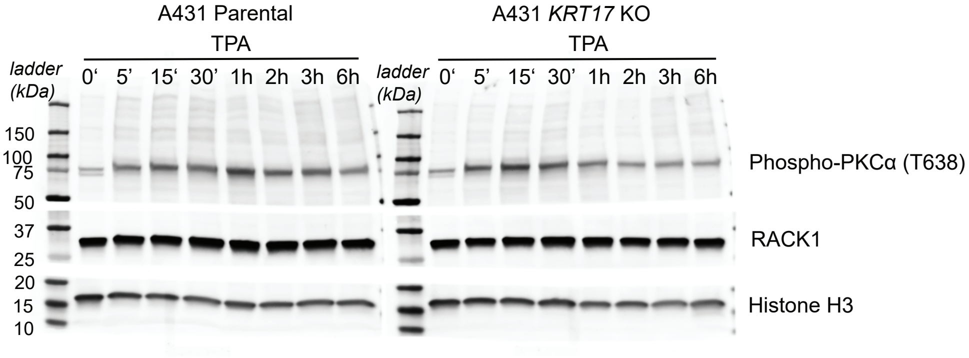

- Samples: Whole cell lysates of A431 parental or A431 *KRT17* KO cells (35 ug total protein/lane).
- Blot was developed using SuperSignal West Pico PLUS chemiluminescent substrate (Thermo Scientific #34580) and imaged using a FluorChem Q system (ProteinSimple) with a 2 min exposure.
- This image was used to genetare Figures 5C-F and 6B.

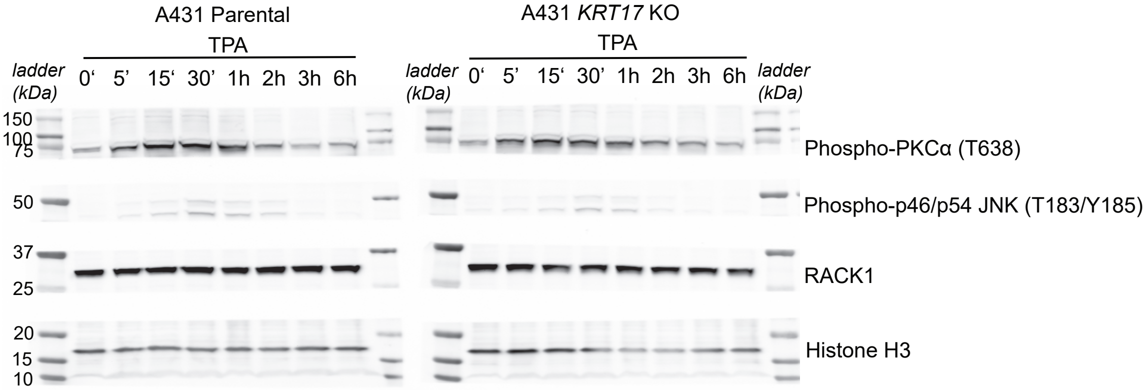

- Samples: Whole cell lysates of A431 parental or A431 *KRT17* KO cells (35 ug total protein/lane).
- Blot was developed using SuperSignal West Pico PLUS chemiluminescent substrate (Thermo Scientific #34580) and imaged using a FluorChem Q system (ProteinSimple) with a 2 min exposure.
- This image was used to genetare Figures 5D-F.

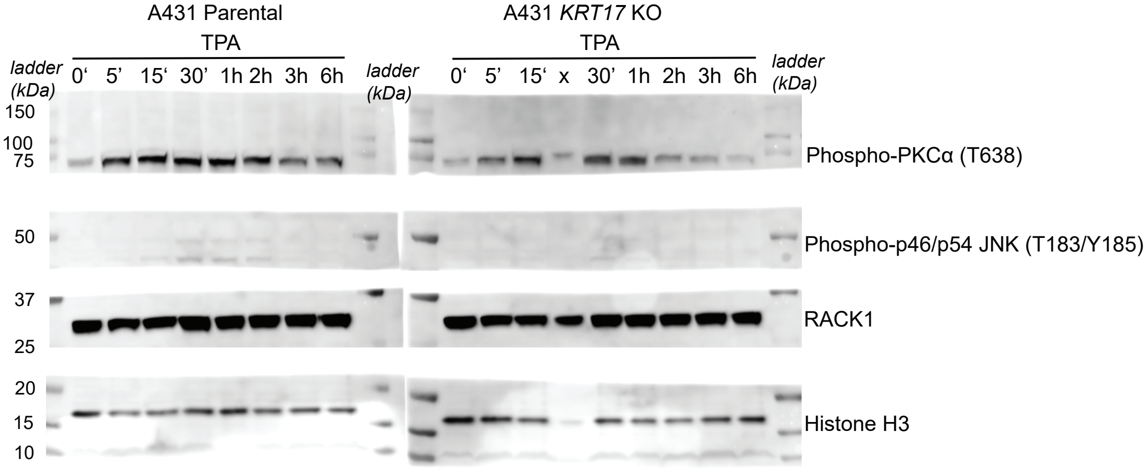

- Samples: Whole cell lysates of A431 parental or A431 *KRT17* KO cells (35 ug total protein/lane).
- Blot was developed using SuperSignal West Pico PLUS chemiluminescent substrate (Thermo Scientific #34580) and imaged using a FluorChem Q system (ProteinSimple) with a 2 min exposure.
- This image was used to genetare Figures 5D-F.

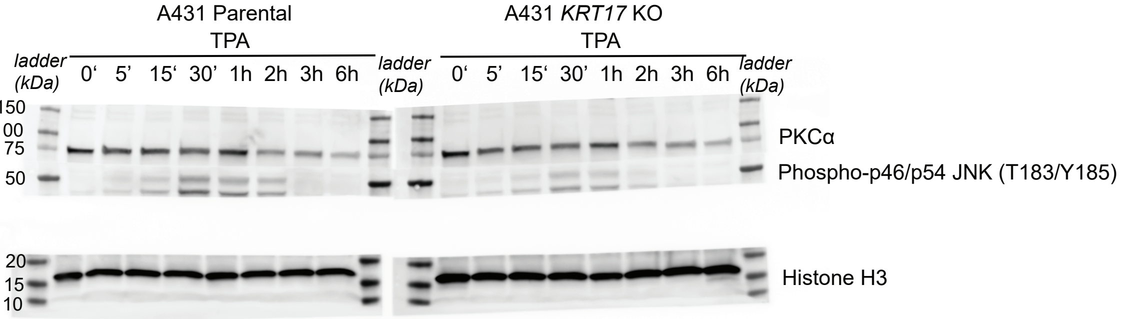

- Samples: Whole cell lysates of A431 parental or A431 *KRT17* KO cells (40 ug total protein/lane).
- Blot was developed using SuperSignal West Pico PLUS chemiluminescent substrate (Thermo Scientific #34580) and imaged using a FluorChem Q system (ProteinSimple) with a 2 min exposure.
- This image was used to genetare Figures 5C-F.

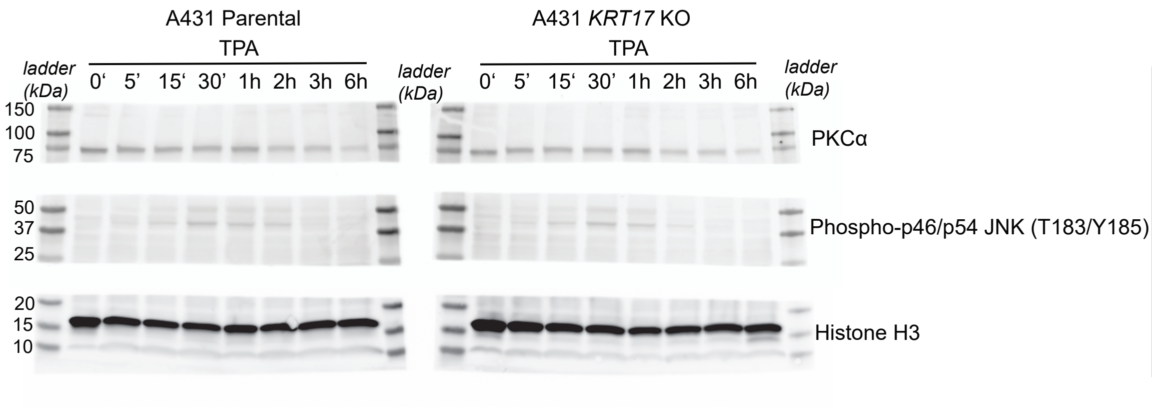

- Samples: Whole cell lysates of A431 parental or A431 *KRT17* KO cells (35 ug total protein/lane).
- Blot was developed using SuperSignal West Pico PLUS chemiluminescent substrate (Thermo Scientific #34580) and imaged using a FluorChem Q system (ProteinSimple) with a 2 min exposure.
- This image was used to genetare Figures 5D-F.

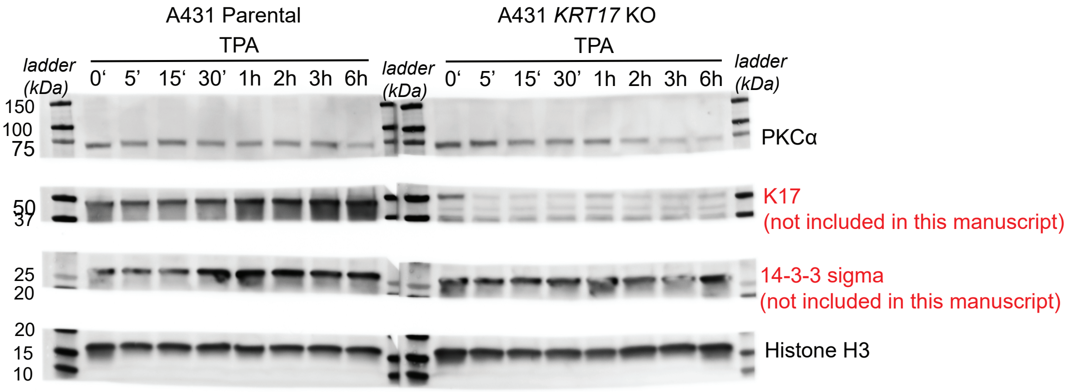

- Samples: Whole cell lysates of A431 parental or A431 *KRT17* KO cells (40 ug total protein/lane).
- Blot was developed using or ECL™ Select Western Blotting Detection Reagent (Cytiva #RPN2235) and imaged using a FluorChem Q system (ProteinSimple) with a 10 sec exposure.
- This image was used to genetare Figures 5D-F.

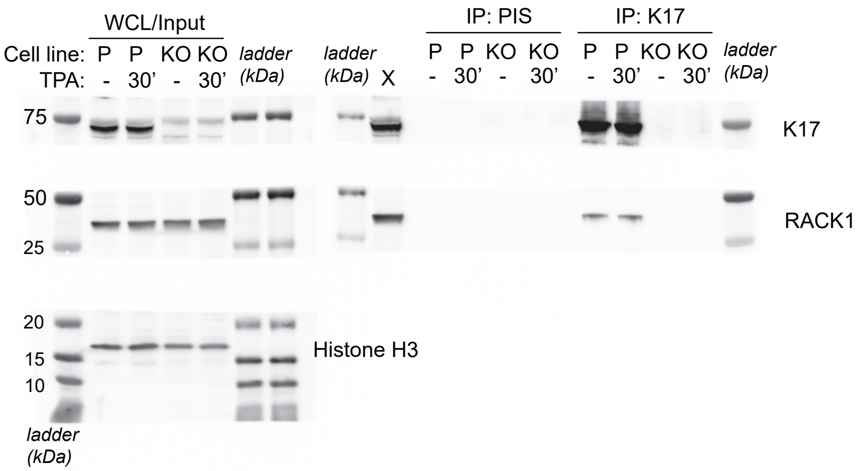

- Samples: 1) Whole cell lysates (WCL) of A431 parental or A431 *KRT17* KO cells (25 ug total protein/lane), and 2) immunoprecipitates (IP) of rabbit preimmune serum (PIS) as IgG control and IP of K17
- Blot was developed using SuperSignal West Pico PLUS chemiluminescent substrate (Thermo Scientific #34580) and imaged using a FluorChem Q system (ProteinSimple) with a 30 sec exposure.
- This image was used to genetare Figure 6A.

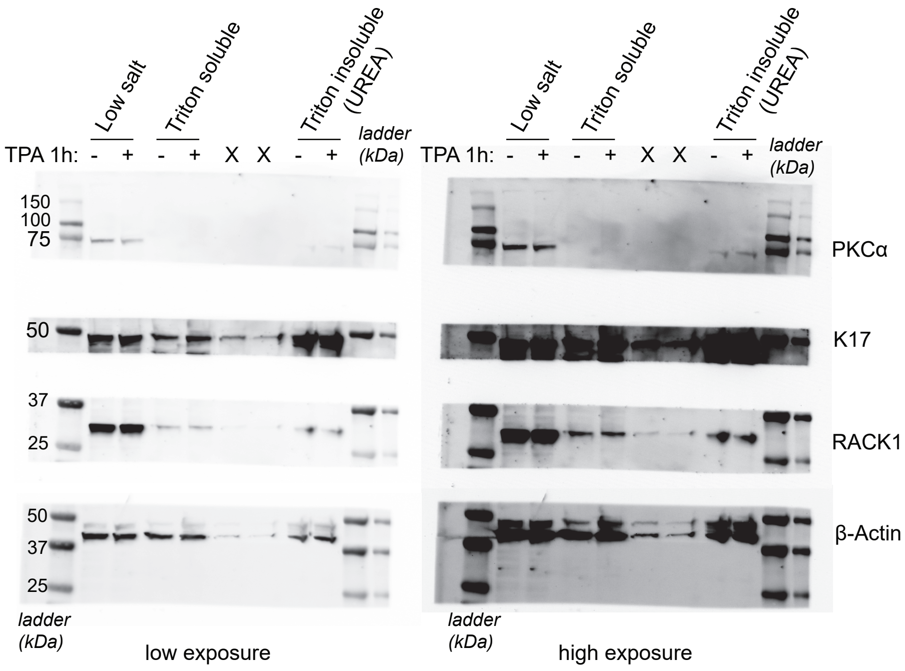

- Samples: Whole cell lysates (WCL) of A431 parental cells.
- Blot was developed using SuperSignal West Pico PLUS chemiluminescent substrate (Thermo Scientific #34580) and imaged using a FluorChem Q system (ProteinSimple) with a 2 min low (left) or 5 min high (right) exposure.
- This image was used to genetare Suppl. Figure 5D.
